# Alkoxyalkylation of Electron-Rich Aromatic Compounds

**DOI:** 10.3390/ijms25136966

**Published:** 2024-06-26

**Authors:** Péter Simon, Bálint Lőrinczi, István Szatmári

**Affiliations:** 1 Institute of Pharmaceutical Chemistry, University of Szeged, Eötvös u. 6, H-6720 Szeged, Hungary; petersimon020@gmail.com (P.S.); lorinczi.balint@szte.hu (B.L.); 2 HUN REN SZTE Stereochemistry Research Group, University of Szeged, Eötvös u. 6, H-6720 Szeged, Hungary

**Keywords:** alkoxyalkylation, alkoxymethylation, hydroxyalkylation, Mannich reaction, *retro*-Mannich reaction, indole, phenol, hydroxyquinoline, quinone methides

## Abstract

Alkoxyalkylation and hydroxyalkylation methods utilizing oxo-compound derivatives such as aldehydes, acetals or acetylenes and various alcohols or water are widely used tools in preparative organic chemistry to synthesize bioactive compounds, biosensors, supramolecular compounds and petrochemicals. The syntheses of such molecules of broad relevance are facilitated by acid, base or heterogenous catalysis. However, degradation of the *N*-analogous Mannich bases are reported to yield alkoxyalkyl derivatives via the *retro*-Mannich reaction. The mutual derivative of all mentioned species are quinone methides, which are reported to form under both alkoxy- and aminoalkylative conditions and via the degradation of the Mannich-products. The aim of this review is to summarize the alkoxyalkylation (most commonly alkoxymethylation) of electron-rich arenes sorted by the methods of alkoxyalkylation (direct or via *retro*-Mannich reaction) and the substrate arenes, such as phenolic and derived carbocycles, heterocycles and the widely examined indole derivatives.

## 1. Introduction

In the field of organic chemistry, C–C coupling reactions are a unique and versatile means of molecular fine-tuning. Coupling a carbon atom bearing active hydrogen with an electron-deficient carbon (e.g., carbonyl) atom has been known to literature. Various methods of synthesizing benzylic-type ethers and alcohols from electron-rich arenes have been introduced using aldehydes as a carbonyl-compound and catalysts such as zeolites, base and (Brönsted or Lewis) acid catalysts or organic amines by the degradation of Mannich bases. These types of products earned significance in pharmaceutical, supramolecular and petrol chemistry, as well as preparative organic chemistry, biosensor chemistry and organic photochemistry.

Regarding alkoxyalkylations, the Mannich or Betti reactions can be regarded as related. The latter reactions can occur via two main mechanisms: one starting with the formation of an iminium ion and its subsequent addition to the electron-rich aromatic ring, the other starting with the formation of a quinone methide and the subsequent *aza*-Michael addition of the amine to the methylidene group [1,2]. Taking the similarities of the hydroxy- or alkoxyalkylation of arenes into consideration, in scientific literature the latter mechanism, starting from quinone methides and the *oxy*-Michael addition of an alcohol, is currently the more frequently proposed mechanism [3,4]. However, considering the fact that alkoxyalkylation is reported as directly using acetals [5,6,7,8], the analogous compounds of imines, the hypothesis of their reaction with aromatic rings should not be neglected.

**Figure 1:**
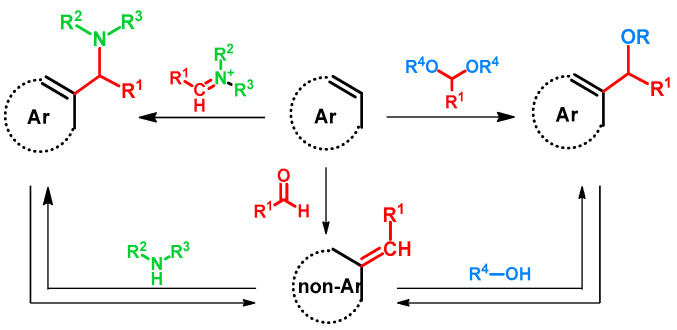
Accepted or experimented reaction pathways of the alkoxymethylation of electron-rich arenes.

More interestingly, as reported, Mannich bases can undergo deaminative reactions yielding quinone methides [1,6,9,10,11,12,13,14,15], from which a broad molecular library can be synthesized of various biological activities. Alkoxyalkyl compounds are also prone to form quinone methides under certain conditions [16], thus opening the way towards aminoalkyl derivatives and a possible synthetic circle. (Figure 1)

The aim of this review is to collect and summarize the aforementioned reactions, focusing on the synthesis of alkoxyalkylated derivatives. The literature processed is grouped with regard to the reaction pathways or substrates, such as the direct alkoxyalkylation via inorganic catalysis or through the Mannich and subsequent *retro*-Mannich reactions.

## 2. Hydroxy- and Alkoxyalkylation of Electron-Rich Arenes: Direct and Catalytic Methods

### 2.1. Hydroxy- and Alkoxymethylation of Monocylic Arenes

**Scheme 1:**
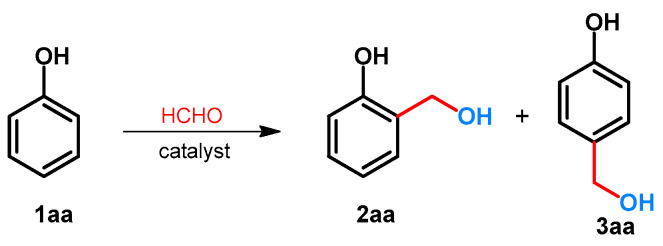
Regioselective catalytic hydroxymethylation of phenol with formaldehyde.

The hydroxymethylation of phenol was examined using formaldehyde. Due to the activation of hydroxyl function, two regioisomeric hydroxymethylated products (**2** and **3**) can be formed. Since both of the products are important chemicals in industry [17,18] or are important precursors toward the synthesis of bioactive compounds [19,20,21], the selectivity of the reaction is critical (Scheme 1). The effect of the divalent metal salts on the regioselectivity was examined by Komiyama and Morozumi [22]. It was found that without catalyst, or by using MgCl_2_, CuCl_2_ and FeCl_2_, the ratio **2aa**/**3aa** was between 1 and 1.5. By applying divalent zinc salts such as Zn(H_3_CCOO)_2_, Zn(NO_3_)_2_ or ZnBr_2_, the selectivity could be shifted toward the formation of **2aa**, and the ratio **2aa**/**3aa** was around 10 [22]. The synthesis of **2aa** was also published by Jeong and Hu[23]. For the synthesis, to provide the selectivity, Zn(NO_3_)_2_.6H_2_O was applied, and 2-hydroxymethylphenol (salicyl alcohol: **2aa**) was further transformed to benzofuran derivatives [23]. 

The prepared salicylic alcohol (**2aa**) was reported as a precursor to synthesizing boron-modified phenolic resin composites (BPR) [24,25]. It was proved that BPR has excellent heat resistance, ablative resistance, good mechanical and wear resistance and flame retardancy [25].

The C-*ortho* site-specific monohydroxymethylation of phenol **1aa** was developed by Casiraghi et al. [26]. For the synthesis of salicyl alcohol **2aa**, phenol **1aa** was treated in xylene with an excess of paraformaldehyde in the presence of 1 mol equiv. of dimethoxyethane (DME) as an ether additive (Table 1, entry 1). The synthetic route was then extended for the synthesis of different salicyl alcohols summarized in Table 1. The *ortho*-selective monohydroxymethylation of a series of phenols was described by Wu et al. [27]. The reactions were performed in the presence of NaBO_2_ at 40 °C. The structures of the synthesized salicyl alcohols are listed in Table 1. It should be mentioned that by using NaBO_2_, the yields were found to be between 80 and 95% (Table 1). Mezzogori et al. reported that the H-mordenite zeolite catalyzed simultaneous synthesis of *o-* (**2ax**), *m-* and *p-*vanillols (Table 1, entry 30) [28]. 

**Scheme 2:**
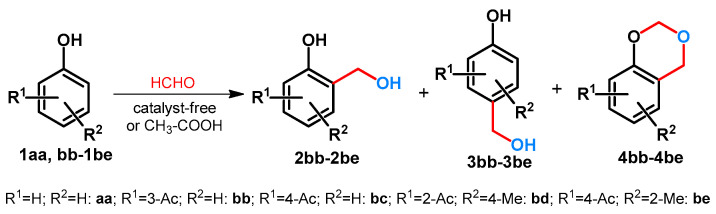
Divergent hydroxymethylation of phenolic compounds.

Hydroxymethylation of phenol (**1aa**) and phenolic ketones (**1bb-1be**) was investigated by Goswami et al. [29]. The reactions were conducted at 60–70 °C. It was found that depending on the phenolic substrate:paraformaldehyde ratios, different products could be isolated. It was also assumed that for the formation of 1,3-dioxane derivatives **4**, the necessary paraformaldehyde ratio depended on the phenol or phenolic ketones (**1**) in question. Starting from **1aa** it was 1:3, from **1bb** and **1bc** 1:7, while from **1be** the ratio was 1:12. It is interesting to note that starting from **1bd**, even by using an extreme excess of paraformaldehyde (1:9), formation of **4bd** was not observed (Scheme 2) [29].

**Scheme 3:**
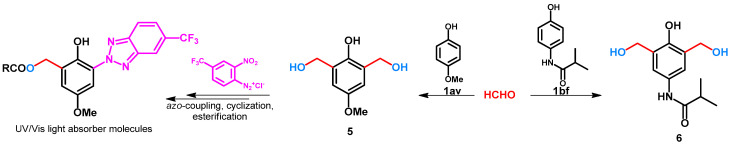
*Ortho*-*ortho’* bis-hydroxymethylation of phenol derivatives.

The previous syntheses, by using different additives or catalysts, attempted to avoid the dihydroxymethylation of phenols. In some cases, the extra hydroxymethyl function can be further used to build bioactive sidechains. For example, the hydroxymethyl group of compound **5** (Scheme 3) was further transformed to benzotriazole moiety [30], while **6** (Scheme 3) was used as an important crosslinking agent [31].

**Scheme 4:**
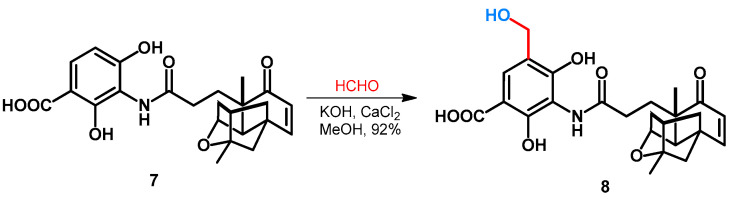
Hydroxymethylation of platensimycin.

Duan et al. reported the hydroxymethylation of platensimycin (**7**) as an excellent natural-product-derived lead molecule against various gram-positive pathogens. The synthesis was performed in MeOH, by using formaldehyde and inorganic additives KOH and CaCl_2_, leading to the formation of **8** in an excellent yield (Scheme 4) [32].

**Scheme 5:**
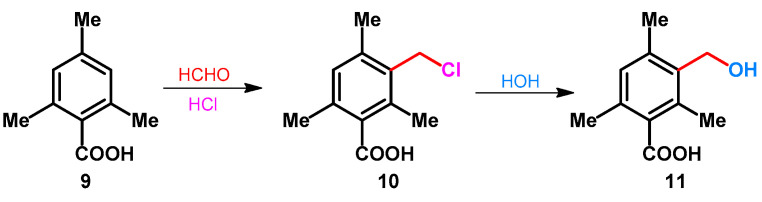
Chloromethylation and rapid hydrolysis of mesitoic acid.

The preparation of 3-hydroxymethyl-2,4,6-trimethylbenzoic acid (**11**) was reported by Stewart. The synthesis included a standard chloromethylation of mesitoic acid (**9**) with formaldehyde in the presence of hydrochloric acid, followed by the rapid hydrolysis of the chloromethyl group during the mildly basic aqueous wash stage incorporated in the work-up procedure (Scheme 5) [33].

**Scheme 6:**
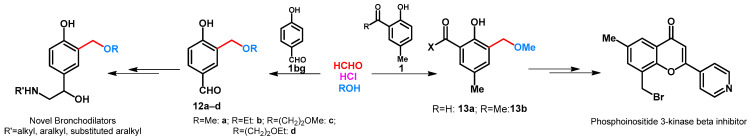
Alkoxymethylation of hydroxybenzaldehydes and phenolic ketones.

The synthesis of 4-hydroxy-3-alkoxymethylbenzaldehydes (**12a-d**) was reported by Hirose et al. The role of hydrochloric acid is to form 3-chloromethyl intermediate that reacts immediately with the alcohols applied in the reaction (Scheme 6) [34]. Methoxymethylation of 2-hydroxy-5-methylbenzaldehyde was published by Jorgensen et al. (Scheme 6) [35]. Compound **13a** was then transformed to new potential fibroblast growth factor (FGF) receptor 1 kinase inhibitors. Methoxymethylation of 2-hydroxy-5-methylacetophenone was performed in methanol by using paraformaldehyde in the presence of cc. hydrochloric acid (Scheme 6) [36]. *Ortho*-situated hydroxyl and acetyl functions enabled the building of substituted pyran moiety.

**Scheme 7:**
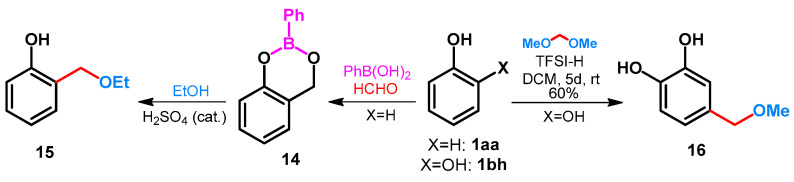
Alkoxymethylation of catechol and phenol with TFSI-H or phenylboronic acid.

*Ortho*-selective ethoxymethylation was published by Bélanger et al. [37]. Phenol **1aa** was reacted with formaldehyde in the presence of phenylboronic acid, leading to 1,3,2-benzodioxaborin **14** (Scheme 7). Treatment of **14** with ethanol in the presence of sulphuric acid catalyst gave the desired 2-ethoxymethylphenol **15**. By treatment of 1,2-dihydroxybenzene (**1bh**) with dimethoxymethane in the presence of bistrifluoromethanesulfonimide (TFSI-H), a regioselective Friedel–Crafts alkylation took place and compound **16** was isolated in a yield of 60% (Scheme 7) [5].

The reaction among resorcinol derivatives and flavor-relevant saturated aldehydes was tested by Zomora et al. [38]. As a representative reaction, the synthesis of 4-(1-hydroxypentyl)-2-methyl-benzene-1,3-diol (**17a**) was carried out at 60 °C. It should be mentioned that thanks to the aliphatic alcohol function, the reaction provided different side products, e.g., methoxylated **17a** (Table 2, entry 1), or by water elimination formation of double bond in the side chain. The asymmetric hydroxyalkylation of different phenols was developed by Casiraghi et al. [39]. The reactions were performed in toluene by using chloroacetaldehyde in the presence of chirally modified aluminum chloride derivatives. It was summarized that chiral Lewis acid promoter and the reactants fine-tuned both the regio- and enantioselectivity of the reaction (Table 2, entries 2–9). Ytterbium(III) trifluoromethanesulfonate (Yb(OTf)_3_)-catalyzed electrophilic aromatic substitution of substituted phenols with ethyl-glyoxylate was published by Wang and Zhang [40]. The reactions were performed at rt, and the lowest yield was observed when the starting phenol derivative was bearing tertiary nitrogen (Table 2, entry 13).

Methoxymethylation of electron-rich monocyclic arenes with formaldehyde-dimethylacetal by using β-Zeolite were performed by Müller et al. First, the method was developed starting from cumol (**18a**) [41], and was then extended to other arenes (**18b–g**) [42]. The structures of methoxymethylated derivatives are summarized in Table 3. 

### 2.2. Hydroxy- and Alkoxymethylation of Phenol-Fused Carbocycles

**Scheme 8:**
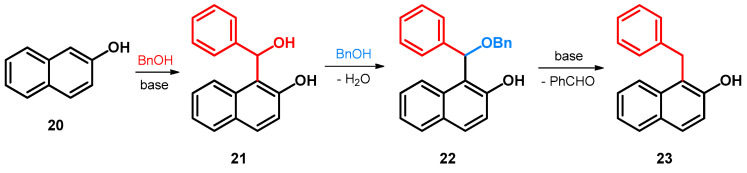
Synthesis of 1-benzylnaphthalen-2-ol (**23**) from 2-naphthol (**20**) and benzyl alcohol.

Regarding alkoxyalkylations of naphthol and its derivatives, the first synthesis concerns such compounds as hydroxylated/alkoxylated bisarylmethylene (BAM) intermediates. In this regard, the reaction of 2-naphthol and benzyl alcohol occurring under high temperature and in the presence of a base—due to which the benzyl alcohol is in situ oxidized to benzaldehyde—yields compound **21**. Subsequently, **21** has been observed to react with the still present benzyl alcohol, yielding compound **22,** which through a base-catalyzed elimination loses benzaldehyde, leaving behind the final benzyl-substituted product **23** (Scheme 8) [43].

**Scheme 9:**
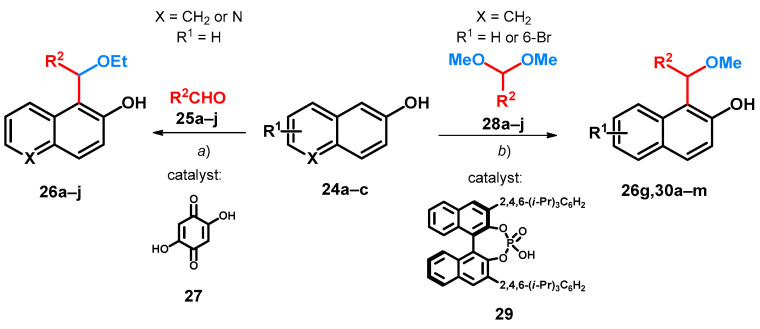
Substituted bisarylmethylene derivatives of 2-naphthol starting from 2-naphthol. (*a*) EtOH/HCl, 100 mol% **27**, r.t., 24 h; (*b*) abs. C_2_H_4_Cl_2_, 20 mol% **29**, 20 mol% AcOH, 35 °C.

First synthesis of alkoxylated BAMs concerns the *oxy*-Michael-type addition reaction between aromatic aldehydes (**25a–j**), EtOH and 2-naphthol (**24a**) or 6-hydroxyquinoline (**24b**) assisted by 2,5-dihydroxy-1,4-benzoquinone (**27**) in the presence of HCl. The reaction afforded 1-[ethoxy(aryl)methyl]-2-naphthol (**26a–g**) and 5-[ethoxy(aryl)methyl]-6-hydroxyquinoline (**26h–j**) derivatives at room temperature (Scheme 9, route *a*, Table 4). The importance of **27** was outlined, as without it only xanthene byproducts could be achieved; however, though it was mentioned as catalyst, highest yields could be achieved at additive concentrations (Entries 6 and 7, Table 5) [44].

Acetals are widely used in carbon–carbon bond-forming reactions with a variety of nucleophiles for the synthesis of ethers [7,8]. Utilizing different chiral BINOL-based phosphoric acids as Brønsted acid catalysts with acetic acid as additive, the Friedel–Crafts-method synthesis of chiral alkoxylated BAMs from various acetals and naphthols (**24a,b**) was also described. After a thorough screening of solvent, catalyst and additive concentration, the chiral phosphoric acid (*R*)-TRIP (**29**) could efficiently catalyze the asymmetric Friedel–Crafts reaction of **24a,b** with different aromatic acetals (**28a–j**), affording chiral ethers (**26g**,**30a–m**) of good enantioselectivity and yield (Scheme 9, Table 6) [6].

It has been previously demonstrated that organic ammonium tribromides (OATB) and in situ generated bromonium ion can be used for *C*–*S* bond cleavage in deprotection of dithioacetals [45] and in hydrolysis of 1-thioglycosides [46]. Starting out from the corresponding unsymmetrical sulfides (**31a–g**)—prepared according to literature methods—the synthesis of alkoxylated BAMs (**32a–r**) or hydroxylated 1-benzylnaphthalen-2-ol (**33s–w**) derivatives could be achieved by utilizing BDMS (bromodimethylsulfonium bromide) and either the corresponding alcohol or water (Table 7) [47].

**Scheme 10:**
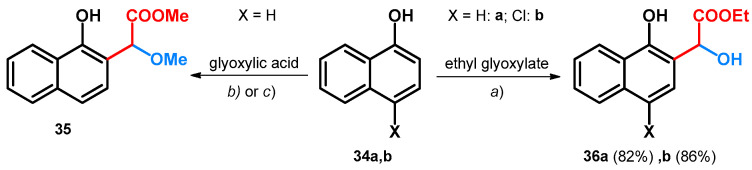
(*a*) NH_2_Boc, *p*TSA, MeOH, reflux, 98 h, 39%; (*b*) acetamide, *p*TSA, MeOH, reflux, 72 h, 34%; (*c*) 5 mol% Yb(OTf)_3_ CH_2_Cl_2_, r.t.

Besides aromatic aldehydes, glyoxylic acid and its esters have also been investigated in the alkoxyalkylation of the electron-rich 1- and 2-napthol systems. Starting out from 1-naphthol (**34a**) and glyoxylic acid, utilizing *tert*-butoxy carbamate or acetamide as catalyst, the methyl 2-methoxyacetate derivative of 1-naphthol (**35**) could be achieved (Scheme 10). Though the syntheses yielded alkoxylated derivatives showing promising catalytic effects in this regard for *tert*-butoxy carbamate and acetamide, the aim of the described work was to synthesize amino acid derivatives through a modified Mannich reaction, thus highlighting the catalysts as unfavorable [48].

When starting out from ethyl glyoxylate and utilizing ytterbium(III) trifluoromethanesulfonate in DCM beside the previously described phenol derivatives (Scheme 10), 1-naphthol (**34a**) and its 4-chloro derivative (**34b**) gave hydroxyl derivatives (**36a**,**b**, Scheme 10) [40].

**Scheme 11:**
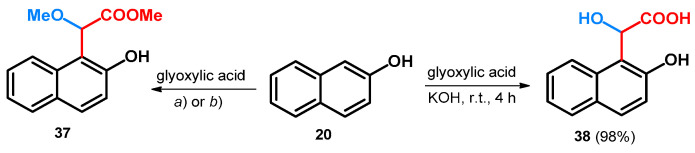
(*a*) NH_2_Boc, PTSA, MeOH, reflux, 93 h, 38%; (*b*) acetamide, PTSA, MeOH, reflux, 84 h, 32%;.

When starting out from 2-naphthol and glyoxylic acid and utilizing the same *tert*-butoxy carbamate or acetamide as catalyst, similar results could be achieved: the methyl 2-methoxyacetate derivative of 2-naphthol (**37**) could be achieved similar to **35** (Scheme 11) [48].

Without the use of such catalysts, applying only KOH, glyoxylic acid condenses with **20**, yielding α-hydroxy-α-(2-hydroxy-1-naphthyl)acetic acid (**38**, Scheme 11). Unfortunately, the acquired enantiomers could not be resolved due to possible racemization under the resolving conditions [49].

**Scheme 12:**
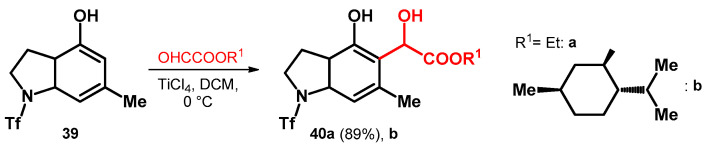
Alkoxyalkylations of pyrrolidine-fused phenol system.

The previously mentioned glyoxylic acid esters have also been utilized on phenols fused with heterocycles. However, in the case of compound **39**—despite the EDGs on the aromatic ring—titanium catalyst was needed most probably due to the strong inactivating effect of the triflate protecting group on the pyrrolidine moieties’ nitrogen. The reaction yielded the ethyl ester derivative **40a** in high yield (89%); however, as far as we know there was no published yield for the relatively more interesting (–)–menthol ester (**40b**, Scheme 12) [50]. 

**Scheme 13:**
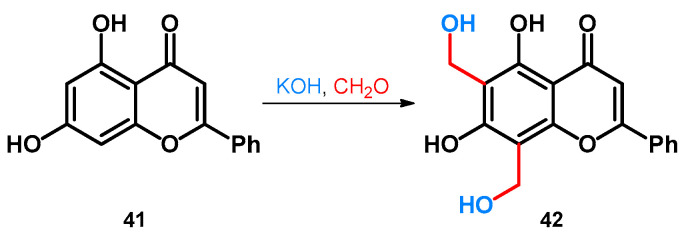
Synthesis of chrysin-based hydroxymethylene derivative **42.**

Regarding phenol-fused ring-systems, another interesting reaction concerns the transformation of chrysin, a naturally occurring flavonoid. The reaction conditions specified in the patent were not discoverable, but similar conditions are also discussed later, in the case of indole derivatives in Section 2.3.1.: the flavonoid (**41**) was reacted with a formaldehyde source in the presence of an alkali hydroxide that gave rise to the formation of the methoxymethylated derivative **42** (Scheme 13) [51].

### 2.3. Hydroxy- and Alkoxymethylation of N-heterocycles

#### 2.3.1. Hydroxy- and Alkoxymethylation of Indoles

**Scheme 14:**
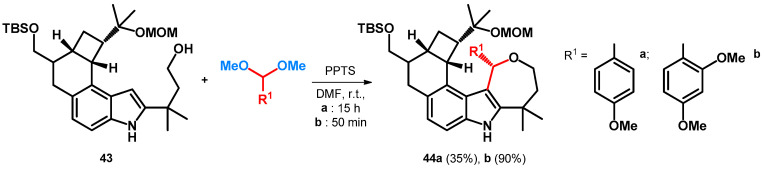
Unsuccessful protection of indole nitrogen yielding BAMs. PPTS = pyridinium *p*-toluenesulfonate, MOM = methoxymethyl ether.

During the development of the total synthesis of penitrems—a family of tremorgenic mycotoxins isolated from *Penicillium crustosum*—researchers were looking for indole nitrogen protecting groups that would not reduce the reactivity of the indole substructure in a modified Mannich reaction. When trying to build in a methoxylated benzyl protecting group, starting out from the acetal of the corresponding aldehydes under acidic conditions with a free alcoholic group—conditions like what later was exploited as described in Section 2.2. Scheme 9—yielded alkoxyalkylated BAMs **44a**,**b** (Scheme 14) [52].

**Scheme 15:**
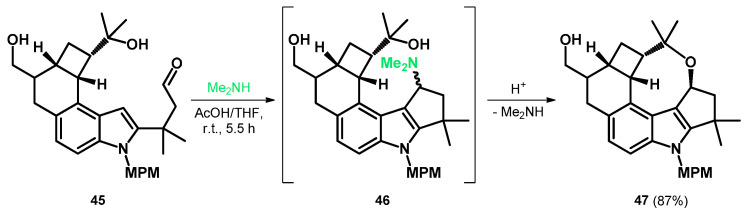
Tandem Mannich cyclization/gramine fragmentation sequence yielding **47.**

Later, MPM (*p*-methoxybenzyl) was found to be a sufficient protecting group and the *N*-protected aldehyde **45** was prepared to take part in the aforementioned modified Mannich reaction, yielding the alkoxyalkylated indole-fused oxocine **47** as the final product via intermediate Mannich-base **46** and a tandem Mannich cyclization/gramine fragmentation sequence (Scheme 15). Subsequently, compound **47** was further transformed to give the A–F rings of penitrems with appropriate functionalization [52].

**Scheme 16:**
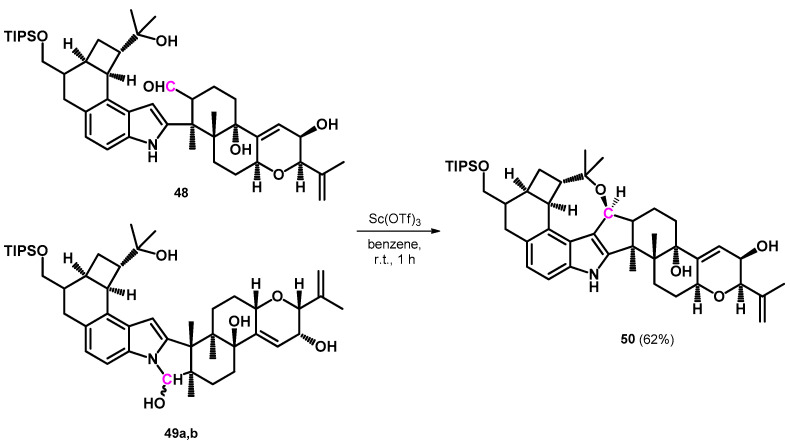
Oxidation of three different intermediates (**48**,**49a**,**b**) into **50** by scandium triflate.

The same research group later described the complete total synthesis of the penitrem ring-systems utilizing a different reaction pathway. This reaction also required the building of the oxocine ring condensed—among other rings—on the indole substructure that required no Mannich-type intermediate. Alcohols **48** and **49a,b** were synthesized and were tandem-cyclized into the oxocine-condensed ring-system (**50**) using scandium triflate (Scheme 16) [53]. 

**Scheme 17:**
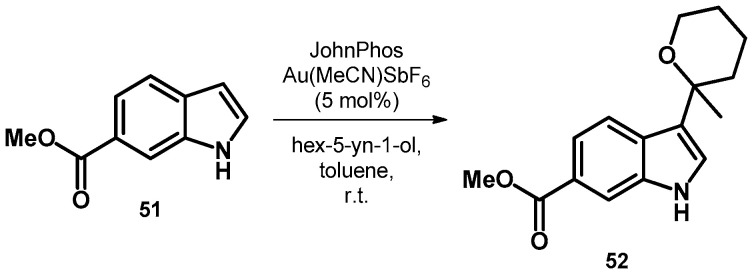
Synthesis of tetrahydro-2*H*-pyran-2-yl-indole derivative **52.**

Starting out from less functionalized indole derivatives, the first publication describes the synthesis of a novel Nav1.7 sodium channel inhibitor discovery. Compound **52** was synthesized uniquely among the other derivatives described using a gold salt as oxidizer starting out from **51** (Scheme 17). Compound **52** was later transformed; however, the final product had no inhibitory effect [54].

Utilizing similar techniques, two research papers describe the *oxy*-Michael-type modification of indole derivatives (**51**,**53a–p**) using aromatic aldehydes and methanol in a similar way as discussed previously in Section 2.2, Scheme 9; however, in these cases the acidic catalysts were exchanged to NaOH still yielding BAMs **54aa–xa** (Table 8) [4,55]. It would be interesting to carry out the previously discussed alterations on 2-naphthols with similar conditions.

Standing out from the transformations discussed previously, a site-selective 1,1-difunctionalization of unactivated alkenes was described that was enabled by cationic palladium catalysis. The scope and limitations of the synthesis were investigated in the case of alkenes comprising alcohols and carboxylic acids (**55a–k**) utilizing palladium(II) acetate and silver hexafluoroantimonate(V) as catalysts yielding **57a–k** (Table 9). It is worth mentioning that for the reaction to take place, directing group **AQ** was necessary and crucial [56].

Another reaction standing out from the ones discussed previously concerns the synthesis of spiro-pyridoindolone derivatives (**59**). However, one similarity ties it to the transformation discussed in Scheme 17, as the reaction concerns an alkyne moiety reacting with an alcohol—intramolecularly in **58**—in the presence of gold salt catalyst (Table 10) [57].

#### 2.3.2. Hydroxy- and Alkoxymethylation of Uracil

Uracil (**60**) bearing an enamino ketone character was successfully utilized in a hydroxyalkylation reaction using electron-deficient aromatic aldehydes in water. Instead of the more commonly used halogeno derivatives of aromatic aldehydes, the scope and limitations were investigated for *p*-nitrobenzaldehyde with heteroaromatic aldehyde derivatives, showing moderate yields (Table 11) [58].

**Scheme 18:**
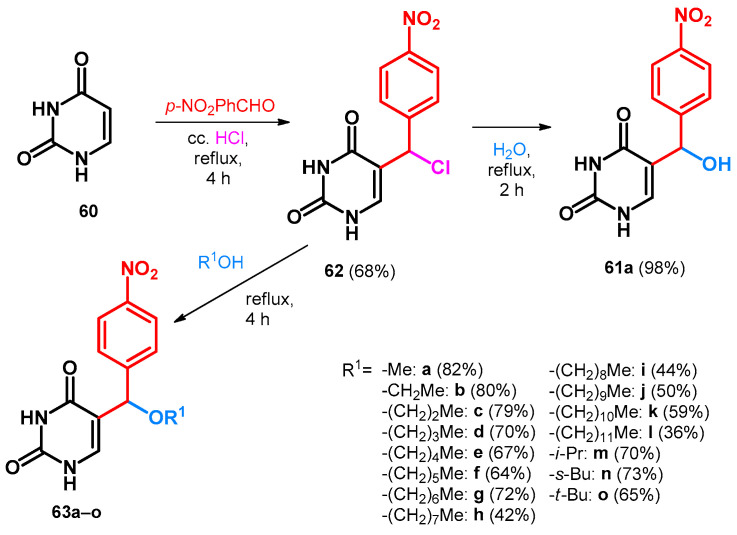
Synthesis of alkoxymethylated BAMs incorporating uracil moiety.

Building upon these transformations, Hlavác et al. described the synthesis of **61a** in very similar reaction conditions. However, instead of the hydroxy derivative, first they obtained the chloro derivative (**62**) that had to be further hydrolyzed without the presence of hydrochloric acid. The scope and limitations was also investigated by supplementing water with different aliphatic alcohols, all yielding the alkoxymethylated BAMs incorporating the uracil moiety (**63a–o**, Scheme 18) [59].

**Scheme 19:**
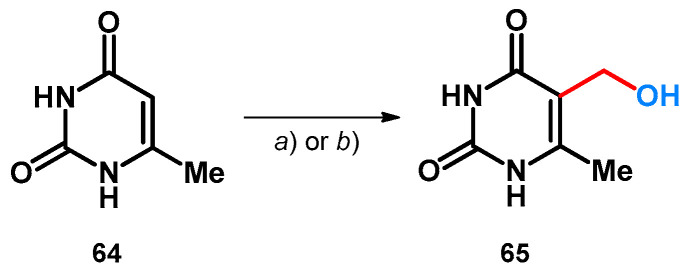
(*a*) (CH_2_O)_n_, KOH, 50 °C, 73 h (76%) [60]; (*b*) (CH_2_O)_n_, KOH, H_2_O, 0 °C then 55 °C for 36 h (98%).

Besides aromatic aldehydes, paraformaldehyde was also investigated in the case of 6-methyluracil. The reaction was carried out using KOH as additive, and in both cases the final product was a hydroxymethylated derivative (**65**). However, it is interesting to mention the differences between the two publications: one describes no solvent and a much longer reaction (route *a*)) [60], while the other describes the solvent and, despite only a 5 °C difference, half the reaction time and much better yields (route *b*)) (Scheme 19) [61]. Both reactions share similarities with the incomplete transformation described in Scheme 13 [51].

## 3. Hydroxy- and Alkoxyalkylation of Electron-Rich Arenes via Aminoalkyl Intermediates

### 3.1. Transformations of Phenolic Mannich Bases

**Scheme 20:**
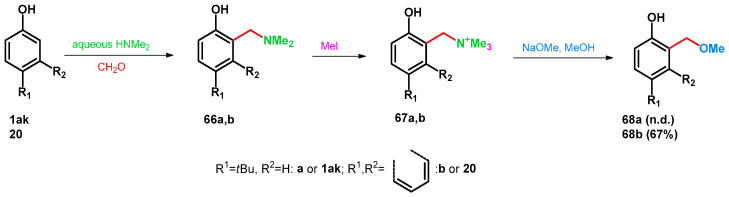
Mannich-subsequent *retro*-Mannich reaction of phenols towards benzyl-methyl ethers.

Transformations of benzylamines with various nucleophiles have been widely investigated applying conditions on these substances, which favor the *retro*-Mannich degradation yield *ortho*-quinone-methides (*o*QM). As these compounds are prone to rearomatization, nucleophilic attack on the methine group leads to novel molecules bearing benzylalcohol or benzyl-alkyl ether fragments. It was published that by the methylation of compounds **66a,b**, unstable quaternary ammonium species formed (**67a,b**) which are easily transformed to benzyl-methyl ethers **68a,b** under treatment with NaOMe in MeOH [62] (Scheme 20).

Based on the findings of Gardner et al., Freccero and his research group in the year 2000 published various transformations of *o*-hydroxybenzyl-trimethylammonium iodide (**69**), testing multiple *N*- (and *S*-) nucleophiles and conditions. UV- and heat-driven eliminative deamination and subsequent nucleophilic addition methods were tested in pH-buffered aqueous solutions [63] (Table 12). 

It was proved that in aqueous conditions, not only is the used *N*-nucleophile capable of the addition towards compounds **70a–e**, but the solvent too can act as a nucleophile yielding salicyl alcohol **2aa**. It was found that via thermodeamination, pH = 6–7 favored the formation of compound **2aa**, while higher pH favored **70a–e**. Using photodeaminative methods (UV 254 nm), the ratio of **2aa** rose higher than formerly in most cases (Table 12). Interestingly, when tyrosine was used, a measurable rate of *O*-alkylation was observed at pH = 10–12 besides the *N*-alkylation.

**Scheme 21:**
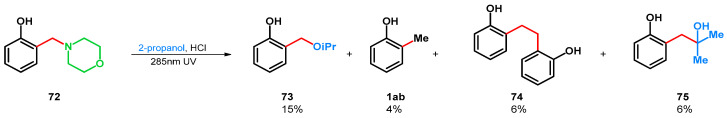
UV-driven *retro*-Mannich reactions of *o*-morpholinomethyl phenol Mannich base.

In 1971 H. J. Roth and K. Michel found that the photodeamination-driven transformation can be applied on tertiary amine **72** in 2-propanol-HCl medium, yielding products **1ab**, **73–75** (Scheme 21) [64]. The radical deamination favored the formation of **73**.

Saito et al. in 1997 published the [65] highly efficient *o*QM formation of dimethylaminomethyl phenols and subsequent transformations toward different acetals. They investigated the *o*QM formation of *o*-hydroxybenzyl alcohols; however, it was found that Mannich bases are much more prone to transformation. 

**Scheme 22:**
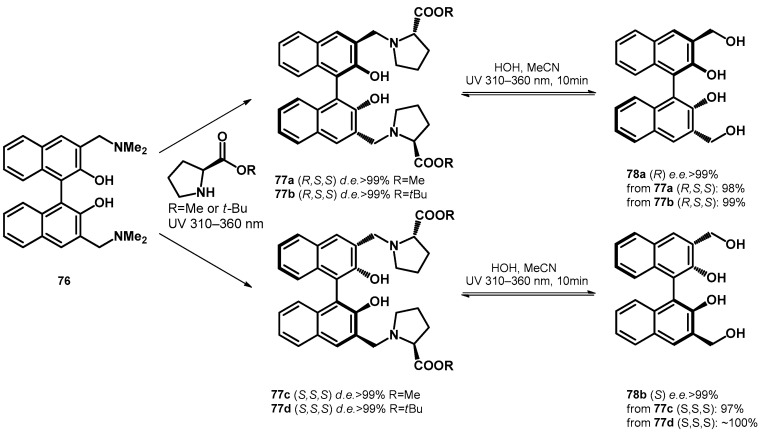
Enantio- and diastereoselective photochemical synthesis of bis-hydroxymethyl BINOLs.

Carrying on the study, Freccero et al. [66] transformed Mannich bases of BINOLs towards L-proline-ester based diastereomers with excellent diastereomeric excess (>99%), which were subsequently transformed to chiral hydroxymethyl derivatives using water/acetonitrile solvent mixture, reaching enantiomeric excess higher than 99%, although they reported the reversibility of the ligand exchange step (**77a–d** and **78a,b**, Scheme 22). 

In the same year, they reported that using thermal incubation or flash photolysis tests, electron-donating substituents facilitate the formation of *o*QMs and suppress the rearomative transformation stabilizing the *o*QM. It can be concluded that electron-withdrawing groups suppress the deamination and facilitate the rearomatization. During flash photolysis test, quaternary ammonium compounds had significantly higher quantum yields than the corresponding tertiary amines (Table 13) [67].

**Scheme 23:**
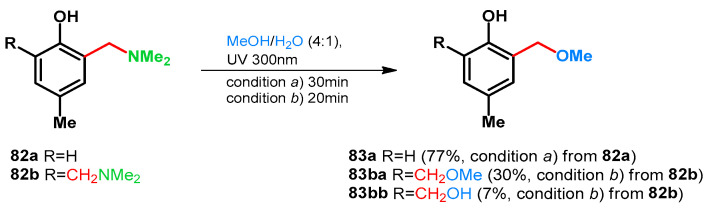
Acid driven photodeamination of tertiary Mannich-base **82a**,**b.**

Basarić et al. in 2015 also supported this while testing the photodeamination of *p*-cresol-derived tertiary amines and their hydrochlorides via laser flash photolysis at 266 nm. It was found that the quantum yield can be elevated by protonation also. (Scheme 23) [68].

**Scheme 24:**
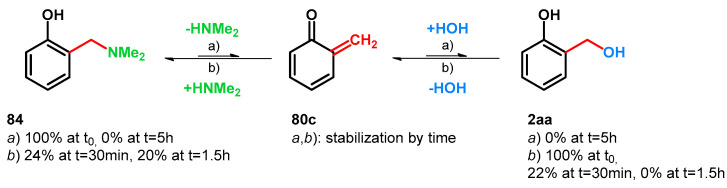
Equilibrium between Mannich-base **84** and salicyl alcohol (**2aa**). Conditions: UV 280 nm, D_2_O/CD_3_CN, Ph_2_CH_2_ (an internal standard); (*a*) **84** 100% at t_0_, HNMe_2_ (40 wt.% in H_2_O, ~1 equiv.); (*b*) **2aa** 100% at t_0_, H_2_O (~1 equiv.).

Takaki et al., while synthetizing chromene derivatives starting from Mannich bases or α-substituted salicyl alcohols, tested the equilibrium of compounds **84, 80c and 2aa**. (Scheme 24)

They also found that in contrast to using quaternary benzylammonium salts, treating secondary Mannich-base **84** with water, UV (>280 nm) in D_2_O/CD_3_CN, no trace of hydrated *o*QM **2aa** was observable (via ^1^H-NMR analysis) (condition *a*); however, starting from **2aa** and reacting it with HNMe_2_ under the same reaction conditions, full conversion was achievable towards **80c** and **84** (condition *b*) [16]. These findings prove the importance of the formation of quaternary ammonium salts of elevated leaving properties regarding *retro*-Mannich transformations. However, elevation of the leaving property of a tertiary amine can be applied through protonation [64,68]. 

**Scheme 25:**
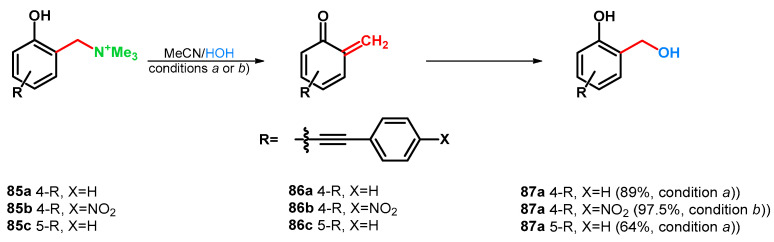
Study on substituent effects in correspondence with *o*QM generation condition (*a*) UV 310nm, 5 min; condition (*b*) UV 360nm, 450 min.

Later, Freccero’s research group broadened the investigations in 2016. Various Mannich bases were synthesized from phenols, bearing 4- or 5-arylethynyl substituents for the investigation of the substituent effect on the photogeneration of *o*QM. An aspect of the *o*QM detection was the study of reactivity with water or mercaptoethanol. Overall findings were that the good-leaving quaternary ammonium moiety is needed for a good quantum-yield photolysis, the 5-arylethynyl derivatives showed lower reactivity and that the electron donating substituents fasten the formation of *o*QMs. Three compounds (**85a–c**) were transformed to hydroxymethyl derivatives as test reactions with water-trapping of **86a–c** (Scheme 25) [69]. 

Basarić et al. in 2020 synthesized fluorescent BODIPY-Mannich dyes which, via *o*QM state, can react with the thiol-groups of BSA (bovine serum albumin). The dyes had strong fluorescence that, in the used concentrations, did not affect cells. Based on the previous studies, hydrochlorides of Mannich bases (**88a,b**) were successfully probed for the transformation with methanol, and the photomethanolysis occurred via irradiation (Table 14) [70]. 

**Scheme 26:**
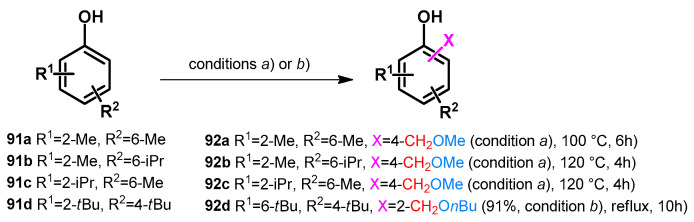
One-pot alkoxymethylation of phenols via subsequent Mannich–*retro*-Mannich reactions: condition (*a*) HNEt_2_ (0.14 equiv.), paraformaldehyde (0.87 equiv.), MeOH; condition (*b*) HN(*n*Bu)_2_ (0.10 equiv.), paraformaldehyde (1.7 equiv.), HOAc (0.5 equiv.), *n*BuOH.

As already stated, polarized *N*-centers favor the *retro*-Mannich transformation towards *o*QMs. Non-UV-driven base and acid catalysis have also been reported to promote such transformations towards *O*-alkylated products. Regarding the synthesis of various chromanecarboxylic acids, the alkoxylmethylation step was reported via the usage of acetic acid additive [71]. However, it was found that a Mannich reaction of disubstituted phenols in alcohols gave alkoxymethylated products while synthesizing antioxidant molecules [72] (Scheme 26).

For the alkoxymethylation of substituted (alkyl, halogeno or *2H*-benzo[*d*][1,2,3]triazol-2-yl) phenols, numerous examples were reported via the treatment of Mannich bases with acetic anhydride and subsequent alkylation with different alcohols. Treating Mannich bases with acetic anhydride yielded *O*-acetyl-*O*-acetyloxymethyl phenols, which under acidic [73] or basic [74] conditions gave the *O*-alkyl products (Table 15). It was found by Crisp and Turner that under mild basic conditions in water, hydroxymethyl derivatives were also able to form, and by the multistep Mannich and subsequent *retro*-Mannich route, in contrast to alkali-mediated hydroxymethylation, higher yields can be achieved (Table 15) [75]. Interestingly, synthesizing benzotriazole derivatives with 2-hydroxyethoxymethyl moieties for step *iii*, 5 equivalents of paraformaldehyde were added. The reason behind this might be that via an in situ acetal formation with ethylene glycol, the formation of bis(benzyloxy)ethylene side-products can be subdued [74].

**Scheme 27:**
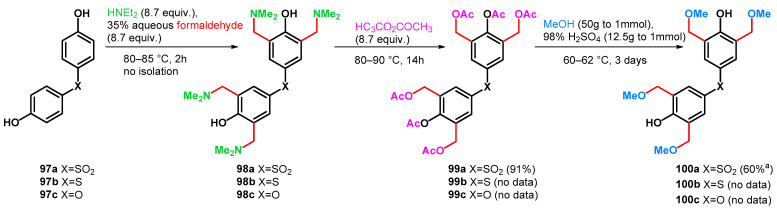
Methoxymethylation of bisphenols **97a**–**c** via Mannich–*retro*-Mannich reactions.

The method itself can be applied to yield tetrakisalkoxymethylated bisphenols as resin raw materials, which have high storage stability and excellent solvent solubility, heat resistance and optical properties [76]. However, it should be noted that from **97a–c** to **99a–c**, multigram scale reactions (0.3 mol of **97a** yielding 0.275 mol of **99a**) were reported, although from **99a–c** to **100a–c**, the data represent a small-scale (0.8 mmol of **99a**) synthesis (Scheme 27).

Alkoxymethylation methods are studied in correspondence with resorcinarene chemistry. Resorcinarenes, which are versatile building blocks for supramolecular chemistry, have been modified yielding alkoxymethylated products. In 2004, Rissanen, Shivanyuk et al. conducted the synthesis of tetrakis(alkoxymethyl)resorcinarenes. Testing reaction conditions, it was found that no conversion was observable without using tromethamine, thus hypothesizing an in situ Mannich reaction by which products transform towards the desired alkoxymethylated products (Table 16) [77]. In 2006, Urbaniak et al. carried on the study using iminodiacetic acid as an *N*-nucleophile. Iminodiacetic acid acts as both *N*-nucleophile and an in situ protonating agent, being an optimal additive for the transformation of electron-rich arenes towards their alkoxymethyl derivatives. It was found that a stoichiometric amount of iminodiacetic acid is not necessary, as a 9.4 mol% catalytic amount was sufficient. They proposed a reaction mechanism which involves autoprotonation of the Mannich-type intermediate [78]. Later, selective alkoxymethylation was carried out using iminodiacetic acid catalyst. They reported selectivity driven by the alteration of the reaction time, as shorter reaction times yielded the less-substituted resorcinarenes (Table 16) [79]. 

### 3.2. Transformations of Semi-Synthetic Phenols and N-heterocycles 

#### 3.2.1. Transformations of Phenol-Fused Molecules

**Scheme 28:**
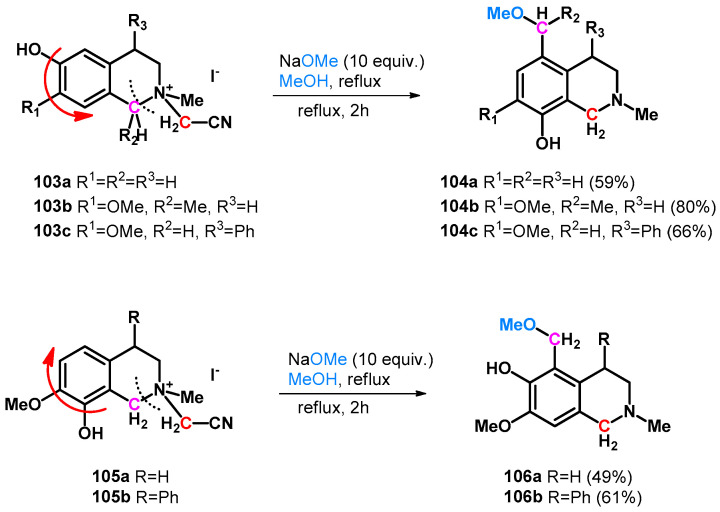
Intramolecular *retro*-Mannich reaction and ring turnover of tetrahydroisoquinolines **103a–c** and **105a**,**b** yielding methoxymethyl derivatives.

Numerous Mannich–*retro*-Mannich-driven hydroxy- and alkoxymethylation reactions have been reported in correspondence with hydroxyfunctionalized heterocycles. In 1995, Hara et al. reported the unexpected methoxymethylation side-reaction of tetrahydroisoquinolines (**103a–c**, **105a**,**b**). The reaction is driven by an intramolecular *retro*-Mannich-type reaction in which *o*- or *p*QM molecules form with simultaneous ring opening and turnover (Scheme 28) [80].

**Scheme 29:**
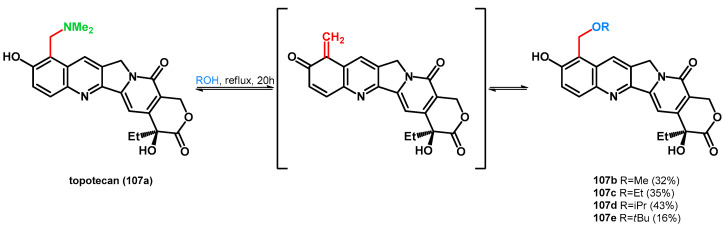
*retro*-Mannich driven *N*–*O* swap of topotecan (**107a**), a widely known Mannich base.

Zhang et al. preformed the alcoholysis of topotecan, the highly water-soluble analogue of 10-hydroxycamptothecine, synthesized via the Mannich reaction [81,82]. The already presented equilibrium was also found between topotecan (**107a**) and compounds **107b–e**, which was explained with the hydrogen bonding ability between the dimethylamino and the phenolic hydroxyl group, thus positively polarizing the nitrogen atom, facilitating the N–C bond cleavage. Compound **106e** showed lower IC_50_ values than the parent molecule on HepG2 and C26 cell lines, presenting the importance of the *N*–*O* swap in pharmaceutical chemistry and drug research (Scheme 29) [83]. Carrying on the study, the same research group broadened the scope of the reaction using thiols and enol-ethers [84].

**Scheme 30:**
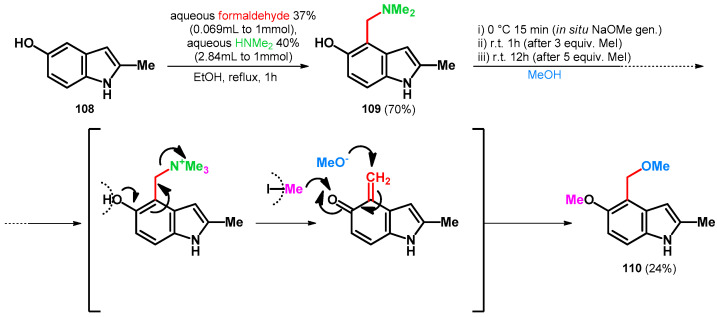
Total synthesis of tricholoma alkaloid **110** utilizing *N*–*O* swap.

Homer and Sperry in 2018 targeted the total synthesis of *Tricholoma* alkaloids [85]. The synthesis started from the Mannich reaction of 5-hydroxy-2-methylindole (**108**) [86] yielding **109,** which was subsequently transformed in one-batch towards **110** with moderate yields (Scheme 30). It is interesting to mention that 6.0 equivalent MeI was added in two portions. It functioned as an *N*-methylating agent [63], facilitating the *retro*-Mannich reaction and also methylating the phenolic hydroxyl group. That compound **110** was isolated with moderate yields might have been caused by the undesired reaction with sodium methoxide and methyl iodide trapping the reagents. The possible product-bearing unmethylated phenolic hydroxyl group was not isolated, as the desired product directly formed in the reaction chamber.

**Scheme 31:**
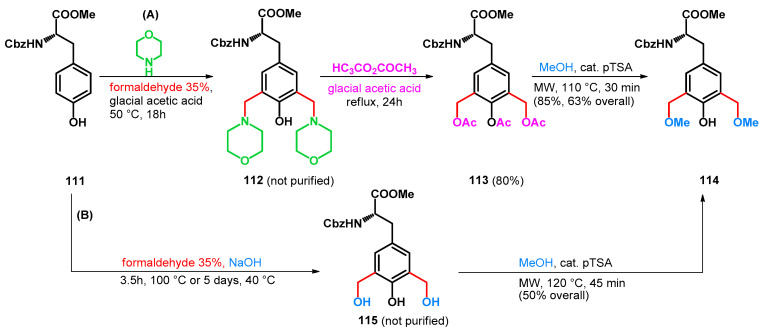
Synthesis of bis-methoxymethyl compound **114**, a building block for methylene-bridged tyrosine derivatives.

In 2006, Bew et al. reported the synthesis of methylene-bridged (*S*)-tyrosine-phenol dimers. While conducting the syntheses, two methods were described, one exploiting the potential of *retro*-Mannich reactions, the other utilizing inorganic additives while reducing reaction steps. Utilizing pathway **A**, slightly higher yields could be achieved than utilizing **B**, despite the greater number of steps and longer reaction time (Scheme 31) [87].

**Scheme 32:**
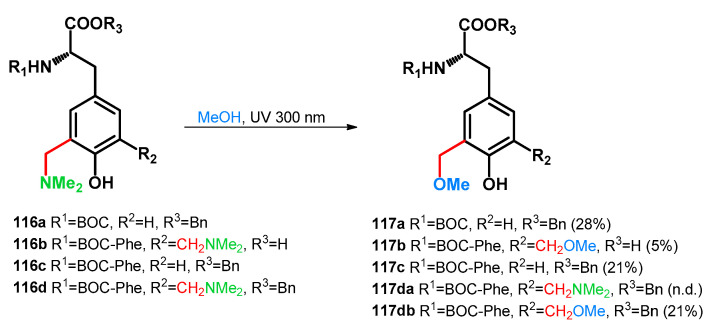
Photochemical synthesis of methoxymethyl tyrosine derivatives and dipeptides.

It is worth mentioning, however, that the usage of amino-acid catalysis (i.e., iminodiacetic acid) has not been tested during the study. In 2006, Basarić et al. reported the photochemical transformations of Mannich bases of dipeptides towards methoxymethylated blocks, presenting photoswitchable units for peptide modification and fine-tuning [88]. Bis(dimethylaminomethyl)-derivative **116d** was investigated in the reaction towards **117da,db**. At 30 min reaction time, the ratio was 1:1, running the reaction for a longer time, 68% conversion towards **117db** was achieved (Scheme 32).

**Scheme 33:**
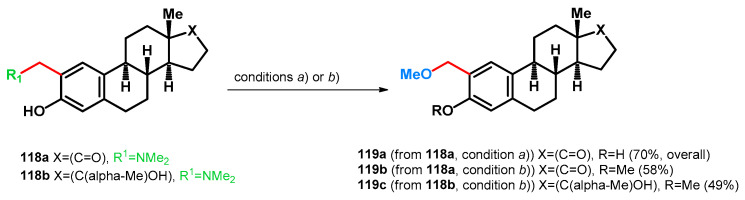
*N*–*O* swap of estrogen-based Mannich bases **118a**,**b** (*a*) (*i*) MeI (26 equiv.), in Et_2_O 20 h, r.t.; isolated (*ii*) KOH (excess), MeOH, 3h, reflux [90] (*b*) MeOH, 17% aqueous KOH (in 2 steps, 12 + 2 weight equiv.), Me_2_SO_4_ (9–10 molar equiv.), 60 °C, 5 h [89].

In patented literature, two research groups have successfully synthesized 2-methoxymethyl estrone or 2-methoxymethyl-17α-estradiol derivatives via different reaction routes starting from Mannich bases **118a–c** [89,90]. It was found that the usage of dimethyl sulfate as an initiator of the *retro*-Mannich subreaction (condition *a*), being a more aggressive alkylating agent than methyl iodide, also alkylated the 3-OH group (**119b**). While using MeI (condition *b*)), as formerly presented, no *O*-alkylation was observed under the investigated conditions. While the latter findings may be advantageous, designing a one-pot method may cause undesired side-reactions, and this should be taken into consideration when conducting alkylative *retro*-Mannich transformations (Scheme 33).

**Scheme 34:**
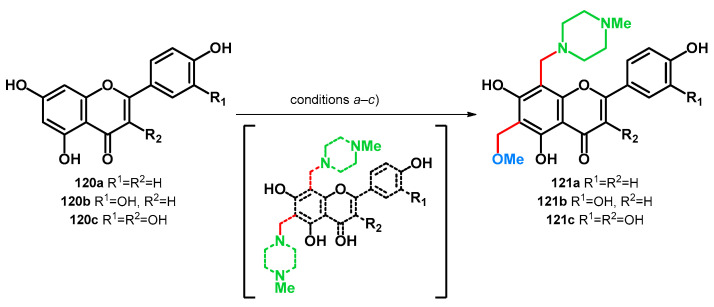
Transformation of flavonoids **120a**–**c** yielding methoxymethyl–morpholinomethyl derivatives: condition (*a*) MeOH, 2.5 equiv. HCHO, 0.8 equiv. *N*-Me-piperazine, 63 °C, 1 h; condition (*b*) MeOH, 1.5 equiv. HCHO, 1.5 equiv. *N*-Me-piperazine, 20 °C, 12 h; condition (*c*) MeOH, 2.0 equiv. HCHO, 1.2 equiv. *N*-Me-piperazine, 46 °C, 6 h.

In 2016, transformations of apigenin (**120a**), luteolin (**120b**) and quercetin (**120c**) via Mannich reaction were reported [91]. It was found that under the investigated conditions (*a*–*c*), running the reaction in MeOH, simultaneous methoxymethylation (at position C–6) and aminomethylation (at position C–8) took place yielding products **121a–c**. Although it has not been investigated, it is noteworthy that a C–6,8 bis-(4-methylpiperazino)methyl derivative is hypothetically possible to form in the reaction mixture. The possible molecules, however, might be prone to transformation towards **121a–c** based on the polarizing properties of the proximal two hydroxyl groups [83] or acidity of the parent molecules themselves [78]; however, the yields have not been reported (Scheme 34).

In 2023, Bereczki et al. synthesized Mannich derivatives of cannabidiol (**122**) in order to enhance their penetrability and/or water solubility [92]. Mannich reaction was an optimal tool; however, conducting the reaction using *n*-buthylamine in ethanol, formation of the ethoxymethylated side-product **124,** in addition to **123**, was observed (condition *b*). The conditions were investigated and optimized, using different solvents (MeOH, 1,4-dioxane) and reaction times. While an *O*-nucleophile alkoxymethylation did not take place in methanol (condition *a*), neither did it using ethanol at lower temperatures (condition *c*) or 1,4-dioxane (condition *d*). The formation of **124** was investigated, and it was found that without an amine component (condition *f*), the desired product did not form. Under condition *e* [38], **124** was isolable with moderate yields (Table 17). 

The information that an equilibrium was found [16] between alkoxymethyl and aminomethyl phenolic compounds, and that product **124** did not form at lower temperatures, suggests that the mentioned equilibrium can be shifted towards the alkoxymethyl compounds by raising temperature. It is notable that higher temperatures might be able to facilitate proton exchange and thus the polarization of the proximal Mannich-*N*-atom.

**Scheme 35:**
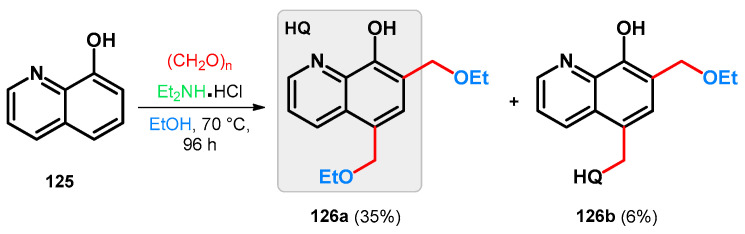
Synthesis of 8-hydroxyquinoline-derived ethoxymethylene derivatives **126a** and **b**.

Modified Mannich reaction of 8-hydroxyquinoline, a widely cited bioactive agent [93,94,95,96,97] (**125**), was reported using diethylamine hydrochloride and paraformaldehyde; however, the isolated products were not identified as Mannich bases. The reaction was carried out in ethanol that most probably served as an *O*-nucleophile on the intermediate Mannich base and exchanged the diethylamino function giving 5,7-bis(ethoxymethyl)quinolin-8-ol (**126a**) as a major product. A dimer (**126b**) was also isolated, formation of which is hypothesized via a *para-*quinone-methide intermediate (Scheme 35) [98].

Taking the aforementioned information into consideration, a hypothetical reason behind the formation of **126a** can be the in situ protonating ability of the diethylamine hydrochloride, thus constituting the driving force of the *retro*-Mannich substep. This is supported by the fact that by utilizing methylene bromide as a methylene source with diethylamine base, the previously targeted diethylaminomethyl-derived 8-hydroxyquinolines were able to be successfully isolated. 

#### 3.2.2. Transformations of N-heterocycles

**Scheme 36:**
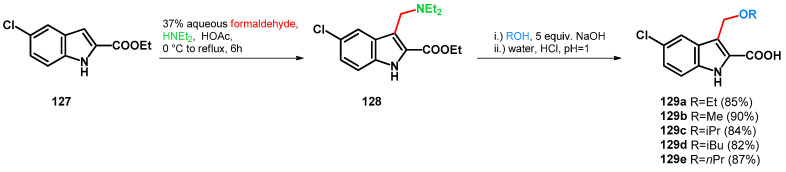
Design of 3-alkoxymethyl indole-2-carboxylic acid antiproliferative agents.

In 2016, Youssif et al. synthesized antiproliferative 3-alkoxymethyl or 3-phenyl indole-2-carboxamides, which showed good activity on MCF7 and HCT116 cell lines (Scheme 36) [99]. In 2022, they carried on the study, synthesizing 2,3-dihydropyrazino [1,2-*a*]indole-1,4-dione derivatives, which showed activity on BRAF^V600E^ and EGFR [100]. It is worth mentioning that in correspondence with both articles, *retro*-Mannich and subsequent alkoxylation reactions were utilized in order to yield the desired products. 

Transformation of **128** towards **129a–e** was conducted in the corresponding alcohols and NaOH with excellent yields. *Retro*-Mannich reaction was reported using both acid and base catalysis in correspondence with the synthesis of indole-based triarylmethanes. The base catalysis is promoted by the deprotonation of the indole and its subsequent deamination at position C–3 [101]. Note, however, that in the case of **129a–e**, theoretically the proximal carboxylic function could act as an autoprotonating agent if the reaction mixture reaches neutral pH and ester function is hydrolized while the aminomethyl moiety is still intact. Also, the *retro*-Mannich reaction can be conducted under acidic conditions present during work-up.

The C–3-alkoxymethyl transformations of kynurenic acid ethyl ester (**130**) were reported by Szatmári et al. [102]. Kynurenic acid being an endogenous neuroprotective agent [103,104,105,106,107,108,109,110,111] of prosperous research, its skeleton has been fine-tuned via the Mannich reaction [112,113,114,115,116,117]. It was found that utilizing secondary amino acid nucleophiles and paraformaldehyde in ethanol, the substrate did not undergo aminomethylation. The isolated product was compound **131a**. Based on previous research, an expansive study was conducted focusing on the optimization of the reaction conditions in order to yield different C–3-alkoxyalkylated kynurenic acid derivatives. Additives triethylamine, NaOEt, pTSA and HOAc did not facilitate the formation of **131a**. Most probably in the case of the electron-deficient arenes (such as **130**), the alkoxyalkylation reaction could be conducted via the Mannich–retro-Mannich route with better conversions.

As a next step for secondary amines, their corresponding acetate salts or amino acid derivatives along with various ampholytic additives were tested in the reaction in order to investigate the simultaneous amino- and alkoxymethylation (Table 18).

It was reported that the low basicity of the nucleophile facilitates the reaction, but it was proposed that using morpholine-based additives, the *O*-atom of the nucleophile might be able to coordinate the solvent, thus elevating the conversion towards **131a,b**. The optimal equivalence was found to be 0.5, able to subdue side-reactions.

The scope of the reaction was investigated using different aldehydes, although no conversion was observed. However, using different alcohols, isopropyl derivative **131b** was isolated. The transesterification and C–3-isopropoxymethylation were found to run simultaneously. Similarly to what was previously reported [92], the desired product did not form using methanol, neither did it using tertiary butanol, which was proposed as being an excessively bulky *O*-nucleophile.

However, the mechanism of the Mannich–*retro*-Mannich-driven alkoxymethylation has been previously proposed [78]. The authors in that case reported indirect proof of the mechanism via the esterification of compound **132c** with ethanol, which instantaneously transformed towards **131a** under the investigated conditions.

## 4. Conclusions and Outlook

The main footsteps of the former years’ research concerning hydroxy- and alkoxyalkylation of electron-rich arenes have been reviewed. First, the focus was pointed towards the direct alkoxy- or hydroxyalkylation of phenols, phenolic ketones and phenol-fused carbo- and heterocycles regarding their hydroxy-functionalized benzene ring, utilizing various catalysts such as sodium metaborate, zeolites as well as homogenous acid and base catalysts. Regioselective hydroxyalkylation has been also successfully conducted. Reviewing the corresponding syntheses, various alkylene sources have been used, such as aldehydes, acetals or, in some cases, dehydrated analogues of oxo-compounds, and alkynes functioned as appropriate substrates. 

In the second part of this review, alkoxy- and hydroxyalkylation via *retro*-Mannich reaction was in focus. The widely used Mannich-reaction, being a versatile tool for C–C bonding, yielded benzylic-type amines using various bioactive compounds (indoles, amino acids and small peptides, cannabinoids, flavonoids, anti-proliferative alkaloids or small heterocycles) as substrates. The Mannich bases already bearing the aminoalkyl moieties were reported to transform towards quinone methides, which compounds subsequently underwent reactions with *O*-nucleophiles. For the quinone methide generation, several methods were grouped, such as photodeamination, *N*-overalkylation, using acid catalysis and *N*–*O* swap via acetyloxylation and subsequent hydro- or alcoholysis. Heterocyclic compounds bearing pyrrol-type nitrogen atoms were reported to undergo such reactions via base catalysis. One-pot methods were also presented in this review, examining the potential of amino-acid catalysis or the driving force of intramolecular proton transfer.

Research focusing on novel C–C bonding formation techniques using water or various alcohols as nucleophiles has already been widely published; however, the recent progress of the field proves its impact.

## Figures and Tables

**Table 1:** *Ortho*-selective hydroxymethylation of substituted phenols using various catalysts.

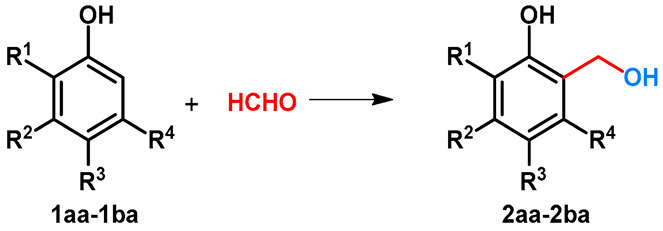								
Entry	R^1^	R^2^	R^3^	R^4^	Product	Conditions	Yield	Reference
1	H	H	H	H	**2aa**	DME/xylene, 135 °C	66	[26]
2	H	H	H	H	**2aa**	NaBO_2_, H_2_O, 40 °C	93	[27]
3	Me	H	H	H	**2ab**	DME/xylene, 135 °C	77	[26]
4	Me	H	H	H	**2ab**	NaBO_2_, H_2_O, 40 °C	87	[27]
5	H	H	Me	H	**2ac**	DME/xylene, 135 °C	92	[26]
6	H	H	Me	H	**2ac**	NaBO_2_, H_2_O, 40 °C	90	[27]
7	H	H	i-Bu	H	**2ad**	DME/xylene, 135 °C	85	[26]
8	Ph	H	H	H	**2ae**	DME/xylene, 135 °C	65	[26]
9	cyclohexyl	H	H	H	**2af**	DME/xylene, 135 °C	85	[26]
10	Me	H	Me	H	**2ag**	DME/xylene, 135 °C	92	[26]
11	H	Me	Me	H	**2ah**	DME/xylene, 135 °C	95	[26]
12	*t*-Bu	H	H	Me	**2ai**	DME/xylene, 135 °C	95	[26]
13	H	H	Ph	H	**2aj**	NaBO_2_, H_2_O, 40 °C	95	[27]
14	H	H	*t*-Bu	H	**2ak**	NaBO_2_, H_2_O, 40 °C	86	[27]
15	H	H	Et	H	**2al**	NaBO_2_, H_2_O, 40 °C	89	[27]
16	F	H	H	H	**2am**	NaBO_2_, H_2_O, 40 °C	85	[27]
17	H	H	F	H	**2an**	NaBO_2_, H_2_O, 40 °C	90	[27]
18	F	F	H	H	**2ao**	NaBO_2_, H_2_O, 40 °C	90	[27]
19	F	H	H	F	**2ap**	NaBO_2_, H_2_O, 40 °C	92	[27]
20	H	H	Cl	H	**2aq**	NaBO_2_, H_2_O, 40 °C	80	[27]
21	H	H	Cl	H	**2aq**	DME/xylene, 135 °C	67	[26]
22	H	Cl	H	H	**2ar**	NaBO_2_, H_2_O, 40 °C	90	[27]
23	H	H	Br	H	**2as**	DME/xylene, 135 °C	69	[26]
24	H	H	Br	H	**2as**	NaBO_2_, H_2_O, 40 °C	83	[27]
25	H	Br	H	H	**2at**	NaBO_2_, H_2_O, 40 °C	91	[27]
26	H	H	I	H	**2au**	DME/xylene, 135 °C	70	[26]
27	H	H	OMe	H	**2av**	DME/xylene, 135 °C	66	[26]
28	H	H	OMe	H	**2av**	NaBO_2_, H_2_O, 40 °C	90	[27]
29	OMe	H	H	H	**2aw**	NaBO_2_, H_2_O, 40 °C	88	[27]
30	OMe	H	H	H	**2ax**	H-mordenite, 80 °C, water/methanol	70	[28]
31	H	H	OBn	H	**2ay**	NaBO_2_, H_2_O, 40 °C	83	[27]
32	H	H	OH	H	**2az**	DME/xylene, 135 °C	59	[26]
33	i-pentyl	H	OH	H	**2ba**	DME/xylene, 135 °C	61	[26]

**Table 2:** *ortho*-hydroxyalkylation of substituted phenols with different catalysts.

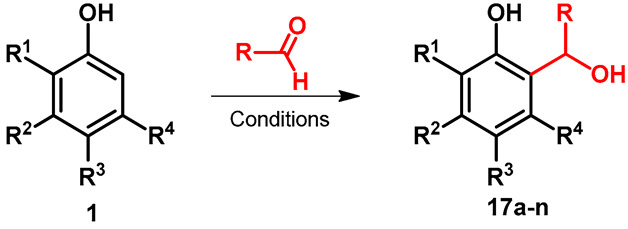									
Entry	R	R^1^	R^2^	R^3^	R^4^	Product	Conditions	Yield	Reference
1	Bu	Me	OH	H	H	**17a**	DME/xylene, 135 °C	66	[38]
2	-CCl_3_	H	H	H	H	**17b**	Toluene, 4 h, 15 °C	96	[39]
3	-CCl_3_	H	H	Me	H	**17c**	Toluene, 4 h, 15 °C	97	[39]
4	-CCl_3_	H	H	*t-*Bu	H	**17d**	Toluene, 4 h, 15 °C	97	[39]
5	-CCl_3_	Me	H	H	H	**17e**	Toluene, 4 h, 15 °C	65	[39]
6	-CCl_3_	i-Pr	H	H	H	**17f**	Toluene, 4 h, 15 °C	78	[39]
7	-CCl_3_	*t*-Bu	H	H	H	**17g**	Toluene, 4 h, 15 °C	53	[39]
8	-CCl_3_	Me	H	H	Me	**17h**	Toluene, 4 h, 15 °C	55	[39]
9	-CCl_3_	Br	H	H	H	**17i**	Toluene, 4 h, 15 °C	51	[39]
10	-COOEt	H	H	*t-*Bu	H	**17j**	Yb(OTf)_3_/CH_2_Cl_2_, 10 h, rt	88	[40]
11	-COOEt	H	*t-*Bu	H	H	**17k**	Yb(OTf)_3_/CH_2_Cl_2_, 10 h, rt	90	[40]
12	-COOEt	H	H	OH	H	**17l**	Yb(OTf)_3_/CH_2_Cl_2_, 10 h, rt	80	[40]
13	-COOEt	H	Et_2_N	H	H	**17m**	Yb(OTf)_3_/CH_2_Cl_2_, 10 h, rt	68	[40]
14	-COOEt	H	H	F	H	**17n**	Yb(OTf)_3_/CH_2_Cl_2_, 10 h, rt	81	[40]

**Table 3:** β-Zeolite-catalyzed *para*-methoxymethylation of electron-rich benzenes.

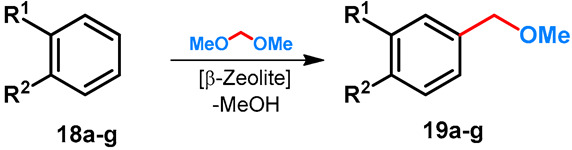						
Entry	R^1^	R^2^	Product	Conditions	Conversion	Reference
1	H	i-Pr	**19a**	17 h, 80–90 °C	12	[41]
2	H	i-Pr	**19a**	54 h, 120 °C	46	[42]
3	H	i-Bu	**19b**	65 h, 75 °C	25	[42]
4	H	*t*-Bu	**19c**	90 h, 70 °C	24	[42]
5	H	OMe	**19d**	6 h, 95 °C	24	[42]
6	OMe	OH	**19e**	3 h, 95 °C	15	[42]
7	OMe	OMe	**19f**	64 h, 95 °C	25	[42]
8	H	OH	**19g**	6 h, 95 °C	24	[42]

**Table 4:** Products and yields of reaction route *a* (check Scheme 9).

X	R^2^	Compound	Yield (%)
CH (**24a**)	*p*-NO_2_C_6_H_4_ (**25a**)	**26a**	86
CH (**24a**)	*m*-NO_2_C_6_H_4_ (**25b**)	**26b**	82
CH (**24a**)	*o*-NO_2_C_6_H_4_ (**25c**)	**26c**	82
CH (**24a**)	*p*-MeC_6_H_4_ (**25d**)	**26d**	79
CH (**24a**)	*p*-(O-CH_2_-Ph)C_6_H_4_ (**25e**)	**26e**	81
CH (**24a**)	*p*-OMeC_6_H_4_ (**25f**)	**26f**	63
CH (**24a**)	2-naphthyl (**25g**)	**26g**	61
N (**24b**)	*p*-BrC_6_H_4_ (**25h**)	**26h**	73
N (**24b**)	Ph (**25i**)	**26i**	71
N (**24b**)	*p*-OMeC_6_H_4_ (**25j**)	**26j**	72

**Table 5:** Catalyst screening of reaction route *a* when X = CH_2_ and R^2^ = *p*-OMe-C_6_H_4_ (check Scheme 9).

Entry	1	2	3	4	6	7
Catalyst (**27**, mol%)	5	25	50	750	100	150
Yield of **28f** (%)	Trace	21	42	63	86	87

**Table 6:** Starting materials, substituents, yields and *ee* for reaction route *b* (check Scheme 9).

24	R^2^	Product	Yield (%)	*ee* (%)
2-naphthol (**24a**)	Ph (**28a**)	**30a**	64	68
2-naphthol (**24a**)	*o*-MeC_6_H_4_ (**28b**)	**30b**	58	55
2-naphthol (**24a**)	*m*-MeC_6_H_4_ (**28c**)	**30c**	61	71
2-naphthol (**24a**)	*p*-MeC_6_H_4_ (**28d**)	**30d**	54	63
2-naphthol (**24a**)	*m*-MeOC_6_H_4_ (**28e**)	**30e**	68	63
2-naphthol (**24a**)	o-ClC_6_H_4_ (**28f**)	**30f**	42	40
2-naphthol (**24a**)	*p*-ClC_6_H_4_ (**28g**)	**30g**	55	58
2-naphthol (**24a**)	*p*-BrC_6_H_4_ (**28h**)	**30h**	51	33
2-naphthol (**24a**)	*p*-FC_6_H_4_ (**28i**)	**30i**	46	50
2-naphthol (**24a**)	2-naphthyl (**28j**)	**30g**	44	66
6-bromo-2-naphthol (**24c**)	Ph (**28a**)	**30j**	57	57
6-bromo-2-naphthol (**24c**)	*m*-MeC_6_H_4_ (**28c**)	**30k**	58	66
6-bromo-2-naphthol (**24c**)	*m*-MeOC_6_H_4_ (**28e**)	**30l**	54	66
1-naphthol (**24c**)	Ph (**28a**)	**30m**	72	20

**Table 7:** Substituted bisarylmethylene derivatives of 2-naphthol starting out from thioethers. (*a*) 2 equiv. R^3^OH, 1 equiv. BDMS, DCM, r.t., 1–2 min. (*b*) 40 μL H_2_O, 1 equiv. BDMS, MeCN, r.t., 5 min.

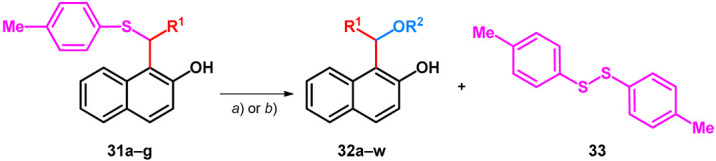				
R^1^	R^2^	Product	Yield (%)	Method
Ph	Et	**32a**	94	(*a*)
Ph	Me	**32b**	90	
Ph	*n*-Pr	**32c**	92	
Ph	i-Pr	**32d**	82	
Ph	*n*-Bu	**32e**	88	
Ph	Bn	**32f**	84	
Ph	CH_2_CH=CH_2_	**32g**	82	
Ph	CH_2_C≡CH	**32h**	80	
Ph	(CH_2_)_3_CH=CH_2_	**32i**	84	
Ph	(CH_2_)_4_Ph	**32j**	88	
Ph	(CH_2_)_2_OH	**32k**	81	
Ph	(CH_2_)_8_OH	**32l**	82	
*p*-NO_2_-Ph	Me	**32m**	94	
*p*-NO_2_-Ph	*n*-Bu	**32n**	88	
*p*-F-Ph	Et	**32o**	94	
*p*-Br-Ph	Me	**32p**	92	
2-C_10_H_7_	Me	**32q**	88	
*p*-Me-Ph	Et	**32r**	90	
Ph	H	**32s**	84	(*b*)
*p*-NO_2_-Ph	H	**32t**	88	
*p*-F-Ph	H	**32u**	86	
*p*-Cl-Ph	H	**32v**	84	
*p*-Me-Ph	H	**32w**	82	

**Table 8:** Alkoxyalkylation of indoles (*a*) 2 equiv. NaOH, 120 °C, 2–24 h; (*b*) 1.5 equiv. NaOH, 20 °C, 24 h.

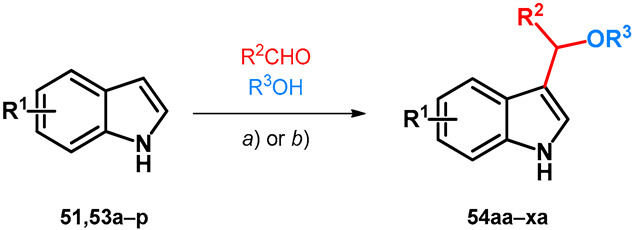									
Products of synthesis route *a*									
R^1^	R^2^	R^3^		Yield (%)	R^1^	R^2^	R^3^		Yield (%)
H	H	Me	**54aa**	89	6-Br	H	Me	**54ap**	80
2-Ph	H	Me	**54ab**	90	7-NO_2_	H	Me	**54aq**	92
4-F	H	Me	**54ac**	88	H	Ph	Me	**54ar**	79
4-Cl	H	Me	**54ad**	82	H	C_5_H_4_N	Me	**54as**	89
4-OMe	H	Me	**54ae**	82	H	*o*-NO_2_C_6_H_4_	Me	**54at**	88
4-OCH_2_Ph	H	Me	**54af**	84	H	*o*-MeC_6_H_4_	Me	**54au**	87
5-Me	H	Me	**54ag**	85	H	*m*- MeC_6_H_4_	Me	**54av**	96
5-OMe	H	Me	**54ah**	81	H	*p*- MeC_6_H_4_	Me	**54aw**	82
5-NO_2_	H	Me	**54ai**	65	H	*m*-CHOC_6_H_4_	Me	**54ax**	96
5-F	H	Me	**54aj**	88	H	*m*-OMeC_6_H_4_	Me	**54ay**	79
5-Cl	H	Me	**54ak**	85	H	*p*-PhC_6_H_4_	Me	**54az**	72
5-Br	H	Me	**54al**	83	H	*p*-CNC_6_H_4_	Me	**54ba**	89
6-COOCH_3_	H	Me	**54am**	57	H	2,4-Cl_2_C_6_H_3_	Me	**54ca**	98
6-F	H	Me	**54an**	81	H	CH_3_CH_3_C_6_H_3_	Me	**54da**	84
6-Cl	H	Me	**54ao**	80	H	H	CH_2_CF_3_	**54ea**	83
Products of synthesis route *b*									
R^1^	R^2^	R^3^		Yield (%)	R^1^	R^2^	R^3^		Yield (%)
H	*p*-CF_3_C_6_H_4_	Me	**54fa**	98	4-Me	*p*-BrC_6_H_4_	Me	**54pa**	89
H	Ph	Me	**54ga**	96	5-Me	*p*-BrC_6_H_4_	Me	**54qa**	92
H	*p*-BrC_6_H_4_	Me	**54ha**	96	6-Me	*p*-BrC_6_H_4_	Me	**54ea**	94
H	*p*-ClC_6_H_4_	Me	**54ia**	95	7-Me	*p*-BrC_6_H_4_	Me	**54sa**	93
H	*p*-FC_6_H_4_	Me	**54ja**	97	5-Cl	*p*-BrC_6_H_4_	Me	**54ta**	96
H	*o*-ClC_6_H_4_	Me	**54ka**	96	5-Br	*p*-BrC_6_H_4_	Me	**54ua**	98
H	3,4-Cl_2_C_6_H_4_	Me	**54la**	50	5-OMe	*p*-CF_3_C_6_H_4_	Me	**54va**	96
H	*p*-NO_2_C_6_H_4_	Me	**54ma**	65	5-CN	*p*-BrC_6_H_4_	Me	**54wa**	42
2-Me	*p*-BrC_6_H_4_	Me	**54na**	96	5-CN	*p*-CF_3_C_6_H_4_	Me	**54xa**	56
2-Me	*p*-ClC_6_H_4_	Me	**54oa**	93					

**Table 9:** Substituents and yields of final products.

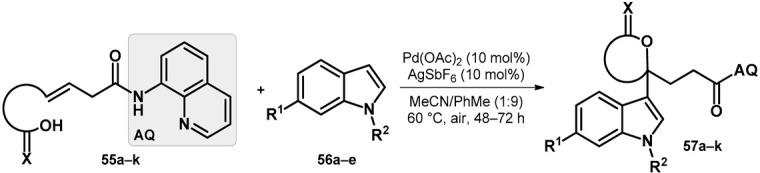									
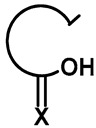	R^1^	R^2^	Compound	Yield (%)	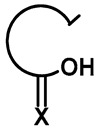	R^1^	R^2^	Compound	Yield (%)
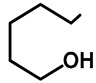	Me	H	**57a**	55	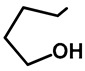	H	F	**57g**	46
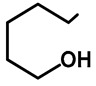	H	H	**57b**	47	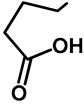	Me	H	**57h**	56 ^a^
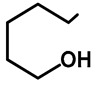	H	OMe	**57c**	56	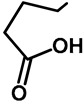	H	H	**57i**	54 ^a^
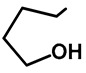	Me	H	**57d**	58	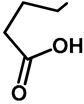	H	OMe	**57j**	56 ^a^
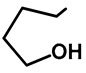	Me	OMe	**57e**	58	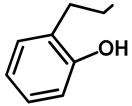	Me	H	**57k**	36
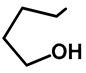	H	H	**57f**	56					

^a^: MeCN was used as solvent at 80 °C.

**Table 10:** Gold-catalyzed intramolecular alkynol cyclization/hydroindolylation.

Reaction Scheme	R^1^	R^2^	Compound	Yield (%)
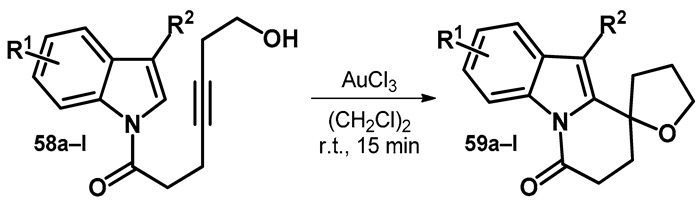	5-OMe	H	**59a**	74
	5-Me	H	**59b**	80
	5-F	H	**59c**	86
	5-Cl	H	**59d**	66
	5-Br	H	**59e**	73
	5-I	H	**59f**	77
	6-F	H	**59g**	90
	6-Cl	H	**59h**	90
	6-Br	H	**59i**	76
	4-Br	H	**59j**	59
	7-Me	H	**59k**	76
	H	2-Me	**59l**	75

**Table 11:** Synthesis and achieved yields of 5-(arylhydroxymethyl)uracil derivatives.

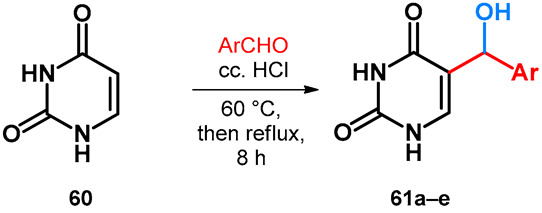					
Ar	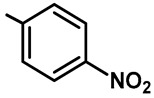	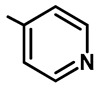	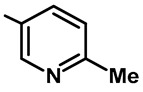	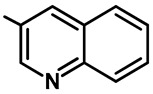	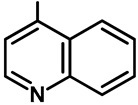
Yield (%)	74	66	70	64	82
Compound	**61a**	**61b**	**61c**	**61d**	**61e**

**Table 12:** Transformations of **69** with amino acids and glutathione as *N*-, *O*- or *S*-nucleophiles.

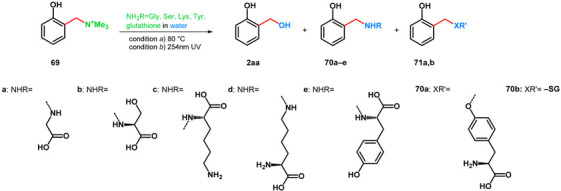				
Product	Condition	NH_2_R	Yield (%)	Maximal Yield (%) of 2aa
**70a**	*a* (pH = 7, 12 h)	glycine	27	70
	*b* (pH = 7, 2 h)		25	75
**70b**	*a* (pH = 6, 15 h)	serine	60	36
	*b* (pH = 7, 2 h)		9	–
**70c,d**	*a* (pH = 5.0, 1 h)	lysine	**c**: 10 (on ω-NH_2_), **d**: 30 (on α-NH_2_)	39
	*b* (pH = 12, 2 h)		**c**: 16 (on ω-NH_2_), **d**: 64 (on α-NH_2_)	–
**70e, 71a**	*a* (1 h)	tyrosine	**e**: max. 64 (pH = 10) **71a**: max. 18 (pH = 12)	max. 32 (pH = 7)
**71b**	*a* (pH = 7, 0.5 h)	glutathione (on thiol)	84	–
	*b* (pH = 7, 2 h)		86	12

**Table 13:** Thermal- and photochemical degradation of Mannich bases **79a**–**k.**

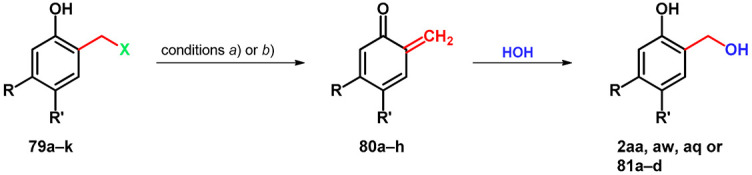						
(*a*) incubation at 100 °C, (*b*) laser flash photolysis, 266 nm						
**Mannich Base**	**X**	**R**	**R’**	**Conditions**	***o*** **QM**	***o*** **-HO-benzylalcohol**
79a	4-morpholinyl	-COOMe	-H	*a*	**80a**	**81a**
79b	4-morpholinyl	-H	-COOMe	*a*	**80b**	**81b**
79c	4-morpholinyl	-H	-H	*a*	**80c**	**2aa**
79d	4-morpholinyl	-H	-OMe	*a*	**80d**	**2aw**
79e	4-morpholinyl	-OMe	-H	*a*	**80e**	**81c**
79f	-N^+^Me_3_	-H	-H	*b*	**80c**	**2aa**
79g	-N^+^Me_3_	-H	-COOMe	*b*	**80b**	**81b**
79h	-N^+^Me_3_	-H	-OMe	*b*	**80d**	**2aw**
79i	-N^+^Me_3_	-H	-Cl	*b*	**80f**	**2aq**
79j	-N^+^Me_3_	-H	-CN	*b*	**80g**	**81d**
79k	-N^+^Me_3_	-H	-CN	*b*	**80h**	**81e**

**Table 14:** Photomethanolysis probe on BODIPY-Mannich dyes.

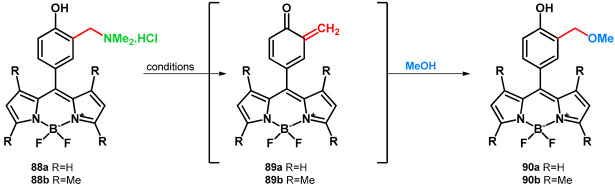					
Unisolated yields of compounds 89a,b and conditions					
#	Solvent	Wavelength of irradiation			
		254 nm	300 nm	350 nm	visible, cool white
**90a**	**MeOH**	decomposed	2	no reaction	
	**MeOH/HOH** 1:1, pH = 7	68 (16 h)	42 (16 h)	2 (16 h)	<1 (16 h)
	**MeOH/HOH** 1:1, pH = 9	decomposed	52 (16 h)	8 (16 h)	<1 (16 h)
**90b**	**MeOH**	30 (9 h)	12 (12 h)	1 (16 h)	no reaction
	**MeOH/HOH** 1:1, pH = 7	100 (2 h)	100 (6 h)	21 (16 h)	2 (16 h)
	**MeOH/HOH** 1:1, pH = 9	94 (4 h)	100 (6 h)	19 (16 h)	2 (16 h)

No data were reported of the summarized isolated yields.

**Table 15:** Mannich and subsequent *retro*-Mannich reaction of substituted phenols via acetyloxylation and hydro- or alcoholysis.

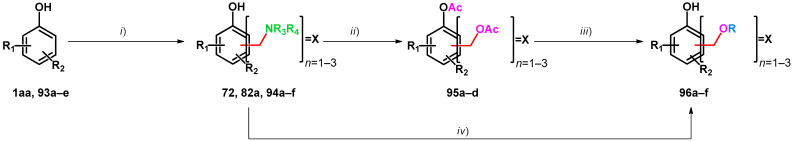								
Compound	Condition (*i*)		Compound	Condition (*ii*)	Compound	Condition (*iii*)	Compound	Ref.
**1aa**: R^1^ = R^2^ = H	10 equiv. 40% formaldehyde in water, 10 equiv. 50% aqueous Me_2_NH, 40 °C, 6 h		**82a**: 2,4,6-X X = CH_2_NMe_2_, R^1^ = R^2^ = H	10 equiv. Ac_2_O, 100 °C, 4 h	**95a**: 2,4,6-X X = CH_2_OAc, R^1^ = R^2^ = H	10. equiv. K_2_CO_3_, EtOH, 80 °C, 2 h	**96a**: 2,4,6-X X = CH_2_OEt	[73]
	2.0 equiv. 38% formaldehyde in water, 0.98 equiv. morpholine, 50 °C, 15 h		**72**: 2,6-X X= morpholino-methyl, R^1^ = R^2^ = H (20%)	Ac_2_O, reflux	**95b**: 2,6-X X = CH_2_OAc, R^1^ = R^2^ = H (57%)	From this step, divergent reaction routes were applied		[75]
**93a**: R^1^ = 4-Br, R^2^ = H	2.2 equiv. 38% formaldehyde in water, 2.2 equiv. morpholine, 80 °C, 6 h, in HOAc		**94a**: 2,6-X X = morpholino-methyl, R^1^ = 4-Br, R^2^ = H (82%)	Ac_2_O (2.2 mL to 1 mol) and HOAc (0.15 to 1 mmol), reflux, 24 h	**95c**: 2,6-X X = CH_2_OAc R^1^ = 4-Br, R^2^ = H (64%)			[75]
**94b** was synthesized via different reaction route			**94b**: 2,6-X X= morpholino-methyl, R^1^ = 4-phenylethynyl	Ac_2_O (11 mL to 1 mmol), HOAc (0.45 to 1 mmol), reflux, 14 h	**95d**: 2,6-X X = CH_2_OAc, R^1^ = 4-phenyl ethynyl, R^2^ = H (75%)	5M H_2_SO_4_ in THF, reflux	**96b**: 2,6-X X = CH_2_OH, R^1^ = 4-phenyl ethynyl, R^2^ = H (48%)	[75]
Compound		Condition (*i*)	Compound		Condition (*iv*)	Compound		Ref.
**93b**: R^1^ = 2-(2*H*-benzo[*d*][1,2,3]triazol-2-yl), R^2^ = Me		50% aqueous Me_2_NH, paraformaldehyde	**94c**: 2-X = CH_2_NMe_2_, R^1^ = 2-(2*H*-benzo[*d*][1,2,3]triazol-2-yl), R^2^ = Me		cat. KOH, 5 equiv. paraformaldehyde ethylene glycol, 150 °C, 24 h	**96c**: 2-X = CH_2_O(CH_2_)_2_OH, R^1^ = 2-(2*H*-benzo[*d*][1,2,3]triazol-2-yl), R^2^ = Me		[74]
**93c**: R^1^ = 2-(2*H*-benzo[*d*][1,2,3]triazol-2-yl), R^2^ = *t-*Bu			**94d**: 2-X = CH_2_NMe_2_, R^1^ = 2-(2*H*-benzo[*d*][1,2,3]triazol-2-yl), R^2^ = *t-*Bu			**96d**: 2-X = CH_2_O(CH_2_)_2_OH, R^1^ = 2-(2*H*-benzo[*d*][1,2,3]triazol-2-yl), R^2^ = *t-*Bu		[74]
**93d**: R^1^ = 2-(2*H*-benzo[*d*][1,2,3]triazol-2-yl), R^2^ = 1-(2-hydroxy)ethyl			**94e**: 2-X = CH_2_NMe_2_, R^1^ = 2-(2*H*-benzo[*d*][1,2,3]triazol-2-yl), R^2^ = 1-(2-hydroxy)ethyl			**96e**: 2-X = CH_2_O(CH_2_)_2_OH, R^1^ = 2-(2*H*-benzo[*d*][1,2,3]triazol-2-yl), R^2^ = 1-(2-hydroxy)ethyl		[74]
**93e**: R^1^ = 2-(2*H*-benzo[*d*][1,2,3]triazol-2-yl), R^2^ = 1-(2,2,4,4-tetramethyl)butyl			**94f**: 2-X = CH_2_NMe_2_, R^1^ = 2-(2*H*-benzo[*d*][1,2,3]triazol-2-yl), R^2^ = 1-(2,2,4,4-tetramethyl)butyl			**96f**: 2-X = CH_2_O(CH_2_)_2_OH, R^1^ = 2-(2*H*-benzo[*d*][1,2,3]triazol-2-yl), R^2^ = 1-(2,2,4,4-tetramethyl)butyl		[74]

**Table 16:** Alkoxymethylation of resorcinarenes using tromethamine (TRIS) or iminodiacetic acid catalyst.

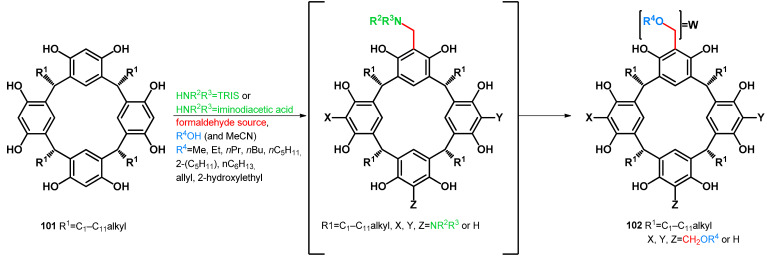			
101	Condition	102	Ref.
**a**: R^1^ = Et	20 equiv. aqueous formaldehyde 37%, HOAc (0.1 mL to 1 mmol), EtOH (10 mL to 1 mmol), 9 equiv. NHR^2^R^3^ = TRIS, 1 h R.T., 1–2 h reflux	**a**: R^1^ = Et, X = Y = Z = W = CH_2_OEt (55%)	[77]
**b**: R^1^ = C_5_H_11_		**b**: R^1^ = C_5_H_11_, X = Y = Z = W = CH_2_OEt (42–69%) ^a^	[77]
**c**: R^1^ = C_11_H_23_		**c**: R^1^ = C_11_H_23_, X = Y = Z = W = CH_2_OEt (42–69%) ^a^	[77]
**d**: R^1^ = i-Bu	5 equiv. aqueous formaldehyde 37%, cat. NHR^2^R^3^ = iminodiacetic acid (in water) (9.4 mol%), R^4^OH as solvent, reflux, 12 h	**d**: R^1^ = i-Bu, X = Y = Z = W = CH_2_OMe (79%)	[78]
		**e**: R^1^ = i-Bu, X = Y = Z = W = CH_2_OEt (66%)	[78]
		**f**: R^1^ = i-Bu, X = Y = Z = W = CH_2_O*n*Pr (64%)	[78]
		**g**: R^1^ = i-Bu, X = Y = Z = W = CH_2_O*n*Bu (46%)	[78]
		**h**: R^1^ = i-Bu, X = Y = Z = W = CH_2_O*n*Pe (40%)	[78]
		**i**: R^1^ = i-Bu, X = Y = Z = W = CH_2_O*n*Hx (28%)	[78]
	2 equiv. aqueous formaldehyde 37%, cat. NHR^2^R^3^ = iminodiacetic acid (10% m/m), R^4^OH as solvent, reflux (reaction times separately assigned)	**j**: R^1^ = i-Bu, X = Y = Z = H, W = CH_2_OMe (34%, 4 h)	[79]
		**k**: R^1^ = i-Bu, X = Y = Z = H, W = CH_2_OEt (39%, 135 min)	[79]
		**l**: R^1^ = i-Bu, X = Y = Z = H, W = CH_2_O*n*Pr (39%, 90 min)	[79]
		**m**: R^1^ = i-Bu, X = Y = Z = H, W = CH_2_OAllyl (43%, 90 min)	[79]
		**n**: R^1^ = i-Bu, X = Y = Z = H, W = CH_2_O(2-hydroxyethyl) (37%, 30 min)	[79]
		**o**: R^1^ = i-Bu, X = Y = H, X = W = CH_2_O*n*Pr (14%, 160 min)	[79]
		**p**: R^1^ = i-Bu, X = Y = H, Z = W = CH_2_O*n*Pr (24%, 160 min)	[79]
		**q**: R^1^ = i-Bu, X = H, Y = Z = W = CH_2_O*n*Pr (29%, 180 min)	[79]
		**r**: R^1^ = i-Bu, X = H, Y = Z = W = CH_2_OAllyl (30%, 180 min)	[79]
**e**: R^1^ = *n*C_11_H_23_	5 equiv. aqueous formaldehyde 37%, cat. NHR^2^R^3^ = iminodiacetic acid (in water) (9.4 mol%), R^4^OH as solvent, reflux, 4 h	**s**: R^1^ = *n*C_11_H_23_, X = Y = Z = W = CH_2_OEt (42%)	[78]
**f**: R^1^ = Me		**t**: R^1^ = Me, X = Y = Z = W = CH_2_OEt (65%)	[78]

^a^ data were not found in experimental data or supporting info.

**Table 17:** Mannich-type modifications of (–)–cannabidiol under different conditions.

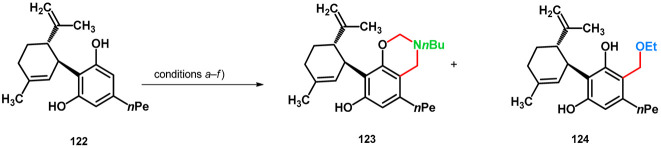			
conditions		**123** (%)	**124** (%)
(*a*)	3.0 equiv. 36% aqueous formaldehyde, 3.0 equiv. NH_2_*n*Bu, MeOH, room temperature, 7 days	89	–
(*b*)	3.0 equiv. 36% aqueous formaldehyde, 3.0 equiv. NH_2_*n*Bu, EtOH, reflux, 20 h	64	21
(*c*)	3.0 equiv. 36% aqueous formaldehyde, 3.0 equiv. NH_2_*n*Bu, EtOH, reflux, 6 h then 45 °C, 18 h overnight	~100	0
(*d*)	3.0 equiv. 36% aqueous formaldehyde, 3.0 equiv. NH_2_*n*Bu, 1,4-dioxane, reflux, 6 h then 50 °C, 18 h overnight	60	–
(*e*)	3.0 equiv. 36% aqueous formaldehyde, 3.0 equiv. NEt_3_, EtOH, reflux, 2 days (reflux, 6 h then 45 °C, 16 h overnight)	–	30
(*f*)	3.0 equiv. 36% aqueous formaldehyde, EtOH, 3 days (reflux, 6 h then 45 °C, 16 h overnight)	–	0 ^a^

^a^ bisarylmethane-type side product formed.

**Table 18:** C–3 alkoxymethylation of kynurenic acid ethyl ester (**130**) via organic additives.

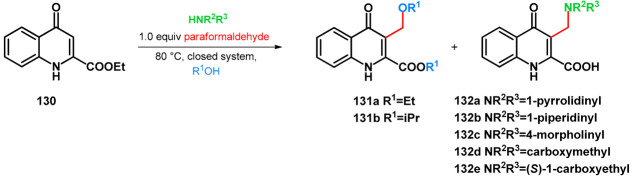				
Conditions			Conversion, Reaction Time (and Isolated Yield)	
HNR^2^R^3^	Equivalence	R^1^OH	131	132
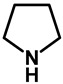	1.0	Ethanol	0%	**a**: 75% (60 h)
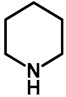	1.0	Ethanol	0%	**b**: 98% (60 h)
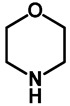	1.0	Ethanol	**a**: 4% (8 h)	**c**: 92% (60 h)
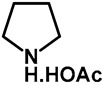	1.0	Ethanol	**a**: 3% (1–3 h)	**a**: 56% (28 h)
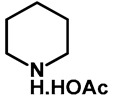	1.0	Ethanol	**a**: 5% (1–5 h)	**b**: 34% (20 h)
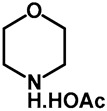	1.0	Ethanol	**a**: 20% (9 h)	**c**: 60% (9 h)
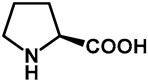	1.0	Ethanol	**a**: 5% (6 h)	0%
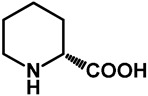	1.0	Ethanol	**a**: 15% (22 h)	0%
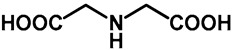	1.0	Ethanol	**a**: 15% (60 h)	0%
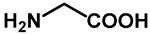	1.0	Ethanol	0%	**d**: isolated 44% (88 h)
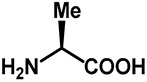	1.0	Ethanol	0%	**e**: isolated 9% (95 h)
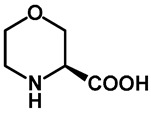	0.1	Ethanol	**a**: 6% (24 h)	0%
	0.5	Ethanol	**a**: 39% (40 h, isolated 33%)	0%
	1.0	Ethanol	**a**: 25% (22 h)	0%
	2.0	Ethanol	**a**: 48% (40 h)	0%
	0.5	2-Propanol	**b**: 21% (26 h, isolated 16%)	0%

## Data Availability

Not Applicable.

## Abbreviations

BAM	bisarylmethylene
BINOL	1,1′-bi-2-naphthol
BDMS	bromodimethylsulfonium bromide
BODIPY	dipyrrometheneboron difluoride
BPR	boron-modified phenolic resin
BSA	bovine serum albumin
DCM	dichloromethane
EDG	electron donating group
MOM	methoxymethyl
MPM	*p*-methoxybenzyl
PPTS	pyridinium *p*-toluenesulfonate
*p*TSA	*p*-toluenesulfonic acid
QM	quinone methide
(*R*)-TRIP	(*R*)-3,3′-bis(2,4,6-triisopropylphenyl)-1,1′-binaphthyl-2,2′-diyl hydrogenphosphate
TBSO	*tert*-butyl-dimethylsilyloxy
TRIS	tris(hydroxymethyl)aminomethane, tromethamine

## References

[1] Barta P., Fülöp F., Szatmári I. (2018). Mannich Base-Connected Syntheses Mediated by *Ortho*-Quinone Methides. Beilstein J. Org. Chem..

[2] Cardellicchio C., Capozzi M.A.M., Naso F. (2010). The Betti Base: The Awakening of a Sleeping Beauty. Tetrahedron Asymmetry.

[3] Li T., Cao M., Liang J., Xie X., Du G. (2017). Mechanism of Base-Catalyzed Resorcinol-Formaldehyde and Phenol-Resorcinol-Formaldehyde Condensation Reactions: A Theoretical Study. Polymers.

[4] Pi C., Yin X., Cui X., Ma Y., Wu Y. (2019). Directed C3-Alkoxymethylation of Indole via Three-Component Cascade Reaction. Org. Lett..

[5] Cossy J., Lutz F., Alauze V., Meyer C. (2002). Carbon-Carbon Bond Forming Reactions by Using Bistrifluoromethanesulfonimide. Synlett.

[6] Qin L., Wang P., Zhang Y., Ren Z., Zhang X., Da C.-S. (2015). Direct Asymmetric Friedel–Crafts Reaction of Naphthols with Acetals Catalyzed by Chiral Brønsted Acids. Synlett.

[7] Zerth H.M., Leonard N.M., Mohan R.S. (2003). Synthesis of Homoallyl Ethers via Allylation of Acetals in Ionic Liquids Catalyzed by Trimethylsilyl Trifluoromethanesulfonate. Org. Lett..

[8] Maity P., Srinivas H.D., Watson M.P. (2011). Copper-Catalyzed Enantioselective Additions to Oxocarbenium Ions: Alkynylation of Isochroman Acetals. J. Am. Chem. Soc..

[9] Szatmári I., Fülöp F. (2011). Simple Access to Pentacyclic Oxazinoisoquinolines via an Unexpected Transformation of Aminomethylnaphthols. Tetrahedron Lett..

[10] Csütörtöki R., Szatmári I., Koch A., Heydenreich M., Kleinpeter E., Fülöp F. (2011). Synthesis and Conformational Analysis of New Naphth[1,2-*e*][1,3]Oxazino[3,4-c]Quinazoline Derivatives. Tetrahedron.

[11] Csütörtöki R., Szatmári I., Koch A., Heydenreich M., Kleinpeter E., Fülöp F. (2012). Syntheses and Conformational Analyses of New Naphth[1,2-*e*][1,3]Oxazino[3,2-c]Quinazolin-13-Ones. Tetrahedron.

[12] Szatmári I., Barta P., Csámpai A., Fülöp F. (2017). Synthesis and Detailed Conformational Analysis of New Naphthoxazino[2,3-a]Benz[c]Azepine and Naphthoxazino[2,3-a]Thieno[3,2-c]Pyridine Derivatives. Tetrahedron.

[13] Szatmári I., Barta P., Tóth G., Balázs A., Halász J., Fülöp F. (2017). Synthesis and Conformational Behaviour of Enantiomeric Naphthoxazinoquinoxalinone Derivatives. Eur. J. Org. Chem..

[14] Szatmári I., Belasri K., Heydenreich M., Koch A., Kleinpeter E., Fülöp F. (2019). *Ortho*-Quinone Methide Driven Synthesis of New *O*, *N*- or *N*,*N*-Heterocycles. ChemistryOpen.

[15] Hegedűs D., Szemerédi N., Spengler G., Szatmári I. (2022). Application of Partially Aromatic *Ortho*-Quionone-Methides for the Synthesis of Novel Naphthoxazines with Improved Antibacterial Activity. Eur. J. Med. Chem..

[16] Fujiwara M., Sakamoto M., Komeyama K., Yoshida H., Takaki K. (2015). Convenient Synthesis of 2-Amino-4 *H* -chromenes from Photochemically Generated *o*-Quinone Methides and Malononitrile. J. Heterocycl. Chem..

[17] Makarov A.S., Kekhvaeva A.E., Chalikidi P.N., Abaev V.T., Trushkov I.V., Uchuskin M.G. (2019). A Simple Synthesis of Densely Substituted Benzofurans by Domino Reaction of 2-Hydroxybenzyl Alcohols with 2-Substituted Furans. Synthesis.

[18] Merkushev A.A., Strel’nikov V.N., Uchuskin M.G., Trushkov I.V. (2017). A Simple Synthesis of Benzofurans by Acid-Catalyzed Domino Reaction of Salicyl Alcohols with N-Tosylfurfurylamine. Tetrahedron.

[19] Choi J., Yeo S., Kim M., Lee H., Kim S. (2018). *P*-Hydroxybenzyl Alcohol Inhibits Four Obesity-related Enzymes in Vitro. J. Biochem. Mol. Toxicol..

[20] Swami Vetha B.S., Adam A.G., Aileru A. (2021). Redox Responsive Copolyoxalate Smart Polymers for Inflammation and Other Aging-Associated Diseases. Int. J. Mol. Sci..

[21] Wendt B., Riem-Ha H., Hesse M. (2002). Synthesis of Two Metabolites of the Antiarrythmicum Amiodarone. Helv. Chim. Acta.

[22] Morozumi T., Komiyama M. (1991). Quantitative Analysis on *Ortho*-Directing Activities of Divalent Metal Salts in Hydroxymethylation of Phenol by Formaldehyde. J. Mol. Catal..

[23] Hu K., Jeong J.-H. (2006). A Convergent Synthetic Study of Biologically Active Benzofuran Derivatives. Arch. Pharm. Res..

[24] Wang F., Huang Z., Liu Y., Li Y. (2017). Novel Cardanol-Containing Boron-Modified Phenolic Resin Composites: Non-Isothermal Curing Kinetics, Thermal Properties, and Ablation Mechanism. High Perform. Polym..

[25] Zhang L., Zhang X., Wang R., Zhang Y., Wu J., Zhou Z., Yin P. (2023). Research Progress in Boron-Modified Phenolic Resin and Its Composites. Polymers.

[26] Casiraghi G., Casnati G., Puglia G., Sartori G. (1980). Selective Reactions between Phenols and Formaldehyde. A Superior Synthesis of Salicyl Alcohols. Synthesis.

[27] Li H.-J., Wu Y.-Y., Wu Q.-X., Wang R., Dai C.-Y., Shen Z.-L., Xie C.-L., Wu Y.-C. (2014). Water-Promoted *Ortho*-Selective Monohydroxymethylation of Phenols in the NaBO_2_ System. Org. Biomol. Chem..

[28] Cavani F., Pozzo L.D., Maselli L., Mezzogori R. (2002). Hydroxymethylation of 2-Methoxyphenol Catalyzed by H-Mordenite: Analysis of the Reaction Scheme. Stud. Surf. Sci. Catal..

[29] Goswami J., Borthakur N., Goswami A. (2003). A Water Based Method for Hydroxymethylation of Phenols and Phenolic Ketones. J. Chem. Res..

[30] Laredo W.R. (2010). UV/Visible Light Absorbers for Ophthalmic Lens Materials. U.S. Patent.

[31] Adegawa Y. (2001). Chemical Amplification Type Negative-Working Resist Composition for Electron Beams or X-rays. Patent.

[32] Tian K., Deng Y., Qiu L., Zhu X., Shen B., Duan Y., Huang Y. (2018). Semisynthesis and Biological Evaluation of Platensimycin Analogues with Varying Aminobenzoic Acids. ChemistrySelect.

[33] Stewart F.H.C. (1981). 3-Chloromethyl-2,4,6-trimethylbenzoic acid. Org. Prep. Proced. Int..

[34] Sohda S., Fujimoto M., Tamegai T., Hirose N. (1979). New Bronchodilators. Synthesis and Bronchodilating Activity of Some 3-(Alkoxymethy1)-a-(N-Substituted Aminomethy1)-4-Hydroxybenzyl Alcohols. J. Med. Chem..

[35] Ravindranathan K.P., Mandiyan V., Ekkati A.R., Bae J.H., Schlessinger J., Jorgensen W.L. (2010). Discovery of Novel Fibroblast Growth Factor Receptor 1 Kinase Inhibitors by Structure-Based Virtual Screening. J. Med. Chem..

[36] Jackson S.P., Robertson A.D., Kenche V., Thompson P., Prabaharan H., Anderson K., Abbott B., Goncalves I., Nesbitt W., Shoenwaelder S. (2004). Inhibition of Phosphoinositide 3-Kinase Beta. Patent.

[37] Lau C.K., Williams H.W.R., Tardiff S., Dufresne C., Scheigetz J., Bélanger P.C. (1989). *Ortho*-Specific Alkylation of Phenols via 1,3,2-Benzodioxaborins. Can. J. Chem..

[38] Hidalgo F.J., Aguilar I., Zamora R. (2017). Model Studies on the Effect of Aldehyde Structure on Their Selective Trapping by Phenolic Compounds. J. Agric. Food Chem..

[39] Bigi F., Cadraghi G., Casnati G., Sartori G., Fava G.G., Belicchi M.F. (1985). Asymmetric Electrophilic Substitution on Phenols. 1. Enantioselective *Ortho*-Hydroxyalkylation Mediated by Chiral Alkoxyaluminum Chlorides. J. Org. Chem..

[40] Zhang W., Wang P.G. (2000). Ytterbium(III) Trifluoromethanesulfonate Catalyzed Electrophilic Aromatic Substitution with Glyoxalate and Lipase-Mediated Product Resolution: A Convenient Route to Optically Active Aromatic r-Hydroxy Esters. J. Org. Chem..

[41] Griesbach U., Mueller U., Puetter H., Quaiser C., Winsel H. (2009). Electrochemical Method for Producing Benzaldehyde Dimethyl Acetylene. Patent.

[42] Griesbach U., Mueller U., Ebel K., Botzem J., Yilmaz B., Stock C. (2009). Method for the Regioselective Alkoxyalkylation of Substituted Benzenes. Patent.

[43] Kito T., Yoshinaga K., Ohkami S., Ikeda K., Yamaye M. (1985). Precursors in the Alkylation of 2-Naphthol with Benzyl Alcohol in the Presence of a Base. J. Org. Chem..

[44] Keshiour S., Shaabani A., Pedarpour M., Sarvary A. (2012). An oxy-Michael addition: 2,5-dihydroxy1,4-benzoquinone-assisted synthesis of 1-[ethoxy(phenyl)methyl]-2-naphthol and 5-[ethoxy(phenyl)methyl]-6-hydroxyquinoline derivatives. Res. Chem. Intermed..

[45] Mondal E., Bose G., Khan A.T. (2001). An Expedient and Efficient Method for the Cleavage of Dithioacetals to the Corresponding Carbonyl Compounds Using Organic Ammonium Tribromide (OATB). Synlett.

[46] Mondal E., Sahu P.R., Khan A.T. (2002). A Useful and Catalytic Method for Protection of Carbonyl Compounds into the Corresponding 1,3-Oxathiolanes and Deprotection to the Parent Carbonyl Compounds. Synlett.

[47] Islam K., Basha R.S., Dar A.A., Das D.K., Khan A.T. (2015). A Direct Approach for the Expedient Synthesis of Unsymmetrical Ethers by Employing Bromodimethylsulfonium Bromide (BDMS) Mediated C-S Bond Cleavage of Naphthalene-2-Ol Sulfides. RSC Adv..

[48] Csütörtöki R., Szatmári I., Mándi A., Kurtán T., Fülöp F. (2011). Synthesis of Hydroxynaphthyl-Substituted α-Amino Acid Derivatives via a Modified Mannich Reaction. Synlett.

[49] Yamaye M., Okano H., Motoyanagi Y., Cho (Toh) N., Yoshinaga T., Tsuru T., Mukae K. (2004). Novel α-Hydroxy- α-(2-Hydroxy-1-Naphthyl)Acetic Acid and Its Derivatives. Preparation and Optical Resolution. Synthesis.

[50] Velthuisen E.J., Weatherhead J.G. (2018). Indoline Derivatives. Patent.

[51] Li F., Zhang Z., Chen L., Wu Z., Wang Z. (2008). 6,8-Dimethylol Chrysin and 6,8-Dimethylol Ether Chrysin, Method for Preparing Same and Pharmaceutical Use. Patent.

[52] Smith A.B., Haseltine J.N., Visncik M. (1989). Tremorgenic Indole Alkaloid Studies 6. Preparation of an Advanced Intermediate for the Synthesis of Penitrem D. Synthesis of an Indole-Oxocane. Tetrahedron.

[53] Smith A.B., Kanoh N., Ishiyama H., Hartz R.A. (2000). Total Synthesis of (−)-Penitrem D. J. Am. Chem. Soc..

[54] Wu Y.-J., Venables B., Guernon J., Chen J., Sit S.-Y., Rajamani R., Knox R.J., Matchett M., Pieschl R.L., Herrington J. (2019). Discovery of New Indole-Based Acylsulfonamide Nav1.7 Inhibitors. Bioorg. Med. Chem. Lett..

[55] Zheng Z., Zha D., Cui P., Zhang H., Li C., Shi J., Han B. (2021). Friedel–Crafts Reaction of Indoles for (3-Indolyl)Methyl Ethers under Basic Condition: Application in Unsymmetrical Bis(Indolyl)Methanes. Results Chem..

[56] Jeon J., Ryu H., Lee C., Cho D., Baik M.-H., Hong S. (2019). Site-Selective 1,1-Difunctionalization of Unactivated Alkenes Enabled by Cationic Palladium Catalysis. J. Am. Chem. Soc..

[57] Shinde M.H., Ramana C.V. (2022). Facile Synthesis of the Spiro-Pyridoindolone Scaffold via a Gold-Catalysed Intramolecular Alkynol Cyclisation/Hydroindolylation. Org. Biomol. Chem..

[58] Lam B.L., Pridgen L.N. (1986). An Acid-Catalyzed Hydroxyalkylation of Uracil: A Facile Synthesis of 5-(Arylhydroxymethyl)Uracils. J. Org. Chem..

[59] Spáčilová L., Džubák P., Hajdúch M., Křupková S., Hradil P., Hlaváč J. (2007). Synthesis and Cytotoxic Activity of Various 5-[Alkoxy-(4-Nitro-Phenyl)-Methyl]-Uracils in Their Racemic Form. Bioorg. Med. Chem. Lett..

[60] Batinac S., Sermek D.M., Cetina M., Pavelić K., Mintas M., Raić-Malić S. (2004). Synthesis of the novel bicyclicoxepino-pyrimidine and fluorinated pyrrolidinopyrimidines. Heterocycles.

[61] Alexander M.D., Chuaqui C., Malona J., McDonald J.J., Ni Y., Niu D., Petter R.C., Singh J. (2016). MK2 Inhibitors and Uses Thereof. U.S. Patent.

[62] Gardner P.D., Rafsanjani H.S., Rand L. (1959). Reaction of Phenolic Mannich Base Methiodides and Oxides with Various Nucleophiles. J. Am. Chem. Soc..

[63] Modica E., Zanaletti R., Freccero M., Mella M. (2001). Alkylation of Amino Acids and Glutathione in Water by *o* -Quinone Methide. Reactivity and Selectivity. J. Org. Chem..

[64] Roth H.J., Michel K. (1971). Bestrahlung von 2-Morpholinomethyl-phenol in Isopropanol 5. Mitt.: Zur Photochemie der 2-Aminomethyl-phenole. Arch. Pharm..

[65] Nakatani K., Higashida N. (1997). Highly Efficient Photochemical Generation of O-Quinone Methide from Mannich Bases of Phenol Derivatives. Tetrahedron Lett..

[66] Colloredo-Mels S., Doria F., Verga D., Freccero M. (2006). Photogenerated Quinone Methides as Useful Intermediates in the Synthesis of Chiral BINOL Ligands. J. Org. Chem..

[67] Weinert E.E., Dondi R., Colloredo-Melz S., Frankenfield K.N., Mitchell C.H., Freccero M., Rokita S.E. (2006). Substituents on Quinone Methides Strongly Modulate Formation and Stability of Their Nucleophilic Adducts. J. Am. Chem. Soc..

[68] Škalamera Đ., Bohne C., Landgraf S., Basarić N. (2015). Photodeamination Reaction Mechanism in Aminomethyl *p* -Cresol Derivatives: Different Reactivity of Amines and Ammonium Salts. J. Org. Chem..

[69] Doria F., Lena A., Bargiggia R., Freccero M. (2016). Conjugation, Substituent, and Solvent Effects on the Photogeneration of Quinone Methides. J. Org. Chem..

[70] Zlatić K., Antol I., Uzelac L., Mikecin Dražić A.-M., Kralj M., Bohne C., Basarić N. (2020). Labeling of Proteins by BODIPY-Quinone Methides Utilizing Anti-Kasha Photochemistry. ACS Appl. Mater. Interfaces.

[71] Hayashibara T., Sato J., Torihara M. (2002). Process for Producing Chroman-Carboxylic Acid. Patent.

[72] Wang Y., Ren H., He S., Yan Y., Wang D., Zhao Z., Wu S., Niu N., Zhang M., Zhang R. (2021). Macromolecular Hindered Phenol Antioxidant and Preparation Method and Application Thereof. Patent.

[73] Kunita K., Aoshima K., Nakamura I., Nakamura T. (1998). Negative Working Image Recording Material. Patent.

[74] Okumuto H., Yamazaki M., Niina N. (2015). Benzotriazole Derivative. Patent.

[75] Crisp G.T., Turner P.D. (2000). Preparation of Functionalised Aryl Alkynes as Precursors to Extended Cyclophanes. Tetrahedron.

[76] Nasu H. (2018). Novel Alkoxymethyl-Substituted Bisphenol Compound. Patent.

[77] Nummelin S., Falabu D., Shivanyuk A., Rissanen K. (2004). Alkoxy-, Acyloxy-, and Bromomethylation of Resorcinarenes. Org. Lett..

[78] Urbaniak M., Iwanek W. (2006). Synthesis of Alkoxymethyl Derivatives of Resorcinarene via the Mannich Reaction Catalysed with Iminodiacetic Acid. Tetrahedron.

[79] Urbaniak M., Pedrycz A., Gawdzik B., Wzorek A. (2013). Preparation of Partially Functionalised Resorcinarene Derivatives. Supramol. Chem..

[80] Hara H., Kaneko K., Endoh M., Uchida H., Hoshino O. (1995). A Novel Ring Cleavage and Recyclization of *N*-Cyanomethyl-1,2,3,4-tetrahydroisoquinolinium Methioidides: A Biomimetic Synthesis of Libetamine. Tetrahedron.

[81] Boehm J.C., Johnson R.K., Hecht S.M., Kingsbury W.D., Holden K.G. (1989). Water Soluble Camptothecin Analogs. Patent.

[82] Bracher F., Tremmel T. (2017). From Lead to Drug Utilizing a Mannich Reaction: The Topotecan Story. Arch. Pharm..

[83] Li J., Wang G., Dong M., Zhang Q. (2011). The Solvolysis of Topotecan in Alcohols and Acetic Anhydride. Bioorg. Med. Chem. Lett..

[84] Tan H., Wang G., Li J., Meng G., Liu Z., Dong M., Li Y., Ju D., Zhang Q. (2015). Synthesis of Novel 10-Hydroxycamptothecin Derivatives Utilizing Topotecan Hydrochloride as *Ortho*-Quinonemethide Precursor. Bioorg. Med. Chem..

[85] Homer J.A., Sperry J. (2018). Synthesis of Three Tricholoma-Derived Indoles via an *Ortho*-Quinone Methide. Arkivoc.

[86] Monti S.A., Johnson W.O. (1970). Position Selective Mannich Reactions of Some 5- and 6-Hydroxyindoles. Tetrahedron.

[87] Bew S.P., Hughes D.L., Sharma S.V. (2006). Acid-Catalyzed Synthesis of Methylene-Bridged (*S*)-Tyrosine−Phenol Dimers. J. Org. Chem..

[88] Husak A., Noichl B.P., Šumanovac Ramljak T., Sohora M., Škalamera Đ., Budiša N., Basarić N. (2016). Photochemical Formation of Quinone Methides from Peptides Containing Modified Tyrosine. Org. Biomol. Chem..

[89] Tanabe M., Peters R.H., Chao W.-R., Shigeno K. (1999). Estrone Sulfamate Inhibitors of Estrone Sulfatase, and Associated Pharmaceutical Compositions and Methods of Use. Patent.

[90] Kaneko H., Hashimoto M., Kawase K. (1964). 2-Methoxymethyl-17alpha-Substituted Estradiol-3-Methyl Ethers and Their Preparation. U.S. Patent.

[91] Meng Z., Jia J., Wang Z., Qiao L., Song J., Yao B., Liang J., Chang Q., Guo Z., Li H.X. (2023). Flavone Derivatives, Preparation Method and Application Thereof. Patent.

[92] Lőrincz E.B., Tóth G., Spolárics J., Herczeg M., Hodek J., Zupkó I., Minorics R., Ádám D., Oláh A., Zouboulis C.C. (2023). Mannich-Type Modifications of (−)-Cannabidiol and (−)-Cannabigerol Leading to New, Bioactive Derivatives. Sci. Rep..

[93] Csuvik O., Szatmári I. (2023). Synthesis of Bioactive Aminomethylated 8-Hydroxyquinolines via the Modified Mannich Reaction. Int. J. Mol. Sci..

[94] Mészáros J.P., Poljarević J.M., Szatmári I., Csuvik O., Fülöp F., Szoboszlai N., Spengler G., Enyedy É.A. (2020). An 8-Hydroxyquinoline–Proline Hybrid with Multidrug Resistance Reversal Activity and the Solution Chemistry of Its Half-Sandwich Organometallic Ru and Rh Complexes. Dalton Trans..

[95] Pape V.F.S., Palkó R., Tóth S., Szabó M.J., Sessler J., Dormán G., Enyedy É.A., Soós T., Szatmári I., Szakács G. (2022). Structure–Activity Relationships of 8-Hydroxyquinoline-Derived Mannich Bases with Tertiary Amines Targeting Multidrug-Resistant Cancer. J. Med. Chem..

[96] Chan S.H., Chui C.H., Chan S.W., Kok S.H.L., Chan D., Tsoi M.Y.T., Leung P.H.M., Lam A.K.Y., Chan A.S.C., Lam K.H. (2013). Synthesis of 8-Hydroxyquinoline Derivatives as Novel Antitumor Agents. ACS Med. Chem. Lett..

[97] Song Y., Xu H., Chen W., Zhan P., Liu X. (2015). 8-Hydroxyquinoline: A Privileged Structure with a Broad-Ranging Pharmacological Potential. Med. Chem. Commun..

[98] Hon Y., Chou Y., Wu I. (2004). Dibromomethane as One-Carbon Source in Organic Synthesis: The Mannich Base Formation from the Reaction of Phenolic Compounds with a Preheated Mixture of Dibromomethane and Diethylamine. Synth. Commun..

[99] Abdelrahman M.H., Aboraia A.S., Youssif B.G.M., Elsadek B.E.M. (2017). Design, Synthesis and Pharmacophoric Model Building of New 3-alkoxymethyl/3-phenyl Indole-2-carboxamides with Potential Antiproliferative Activity. Chem. Biol. Drug Des..

[100] Gomaa H.A.M., Shaker M.E., Alzarea S.I., Hendawy O.M., Mohamed F.A.M., Gouda A.M., Ali A.T., Morcoss M.M., Abdelrahman M.H., Trembleau L. (2022). Optimization and SAR Investigation of Novel 2,3-Dihydropyrazino[1,2-a]Indole-1,4-Dione Derivatives as EGFR and BRAFV600E Dual Inhibitors with Potent Antiproliferative and Antioxidant Activities. Bioorg. Chem..

[101] Deb M., Das C., Deka B., Saikia P., Baruah P. (2016). Hydrogen-Bond-Catalyzed Arylation of 3-(Aminoalkyl)Indoles via C–N Bond Cleavage with Thiourea under Microwave Irradiation: An Approach to 3-(α,α-Diarylmethyl)Indoles. Synlett.

[102] Simon P., Lőrinczi B., Szatmári I. (2024). C–3 Alkoxymethylation of 4-Oxo-1,4-Dihydroquinoline 2-Carboxylic Acid Esters via Organic Additives. Heliyon.

[103] Zádori D., Veres G., Szalárdy L., Klivényi P., Toldi J., Vécsei L. (2014). Glutamatergic Dysfunctioning in Alzheimer’s Disease and Related Therapeutic Targets. J. Alzheimers Dis..

[104] Zádori D., Nyiri G., Szőnyi A., Szatmári I., Fülöp F., Toldi J., Freund T.F., Vécsei L., Klivényi P. (2011). Neuroprotective Effects of a Novel Kynurenic Acid Analogue in a Transgenic Mouse Model of Huntington’s Disease. J. Neural Transm..

[105] Rózsa É., Robotka H., Vécsei L., Toldi J. (2008). The Janus-Face Kynurenic Acid. J. Neural Transm..

[106] Sas K., Robotka H., Toldi J., Vécsei L. (2007). Mitochondria, Metabolic Disturbances, Oxidative Stress and the Kynurenine System, with Focus on Neurodegenerative Disorders. J. Neurol. Sci..

[107] Gigler G., Szénási G., Simó A., Lévay G., Hársing L.G., Sas K., Vécsei L., Toldi J. (2007). Neuroprotective Effect of L-Kynurenine Sulfate Administered before Focal Cerebral Ischemia in Mice and Global Cerebral Ischemia in Gerbils. Eur. J. Pharm..

[108] Kiss C., Shepard P.D., Bari F., Schwarcz R. (2004). Cortical Spreading Depression Augments Kynurenate Levels and Reduces Malonate Toxicity in the Rat Cortex. Brain Res..

[109] Wielosz M., Turski W.A., Urbañska E.M. (2003). Endogenous level of kynurenic acid and activities of kynurenine aminotransferases following transient global ischemia in the gerbil hippocampus. Pol. J. Pharmacol..

[110] Nilsson L.K., Linderholm K.R., Engberg G., Paulson L., Blennow K., Lindström L.H., Nordin C., Karanti A., Persson P., Erhardt S. (2005). Elevated Levels of Kynurenic Acid in the Cerebrospinal Fluid of Male Patients with Schizophrenia. Schizophr. Res..

[111] Fejes A., Párdutz A., Toldi J., Vécsei L. (2011). Kynurenine Metabolites and Migraine: Experimental Studies and Therapeutic Perspectives. Curr. Neuropharmacol..

[112] Nagy K., Plangár I., Tuka B., Gellért L., Varga D., Demeter I., Farkas T., Kis Z., Marosi M., Zádori D. (2011). Synthesis and Biological Effects of Some Kynurenic Acid Analogs. Bioorg. Med. Chem..

[113] Tiszlavicz Z., Németh B., Fülöp F., Vécsei L., Tápai K., Ocsovszky I., Mándi Y. (2011). Different Inhibitory Effects of Kynurenic Acid and a Novel Kynurenic Acid Analogue on Tumour Necrosis Factor-α (TNF-α) Production by Mononuclear Cells, HMGB1 Production by Monocytes and HNP1-3 Secretion by Neutrophils. Naunyn-Schmiedebergs Arch. Pharmacol..

[114] Gellért L., Fuzik J., Göblös A., Sárközi K., Marosi M., Kis Z., Farkas T., Szatmári I., Fülöp F., Vécsei L. (2011). Neuroprotection with a New Kynurenic Acid Analog in the Four-Vessel Occlusion Model of Ischemia. Eur. J. Pharm..

[115] Marosi M., Nagy D., Farkas T., Kis Z., Rózsa É., Robotka H., Fülöp F., Vécsei L., Toldi J. (2010). A Novel Kynurenic Acid Analogue: A Comparison with Kynurenic Acid. An in Vitro Electrophysiological Study. J. Neural Transm..

[116] Vámos E., Párdutz Á., Varga H., Bohár Z., Tajti J., Fülöp F., Toldi J., Vécsei L. (2009). L-Kynurenine Combined with Probenecid and the Novel Synthetic Kynurenic Acid Derivative Attenuate Nitroglycerin-Induced nNOS in the Rat Caudal Trigeminal Nucleus. Neuropharmacology.

[117] Lőrinczi B., Csámpai A., Fülöp F., Szatmári I. (2020). Synthesis of New C-3 Substituted Kynurenic Acid Derivatives. Molecules.

